# Copper Nanoclusters with Exposed Active Sites for Highly Efficient Protoboration of C–C Multiple Bonds

**DOI:** 10.3390/molecules31172956

**Published:** 2026-08-24

**Authors:** Shaoke Zhang, Songwei Wen

**Affiliations:** 1 Advanced Institute for Ocean Research, Southern University of Science and Technology (SUSTech), Shenzhen 518055, China; 2 Department of Chemistry, Southern University of Science and Technology (SUSTech), Shenzhen 518055, China

**Keywords:** copper nanoclusters, protoboration, organoboron compounds, low catalyst loading, stable recyclability

## Abstract

Although atomically precise metal nanoclusters bridge homogeneous and heterogeneous catalysis, their application in the protoboration of C–C unsaturated bonds remains challenging. Herein, we report a stable copper nanocluster (**Cu_6_-1**) featuring exposed active sites, which successfully enables efficient protoboration of alkynes. This catalyst can be readily synthesized on a gram scale and exhibits ultrahigh catalytic efficiency under ambient conditions (room temperature and air atmosphere), achieving a turnover number (TON) of up to 32,000. The reaction demonstrates excellent regio- and stereoselectivity, and the substrate scope has been successfully expanded to alkenes and enynes. More importantly, **Cu_6_-1** overcomes the recyclability issues of traditional catalysts, retaining its activity over eight consecutive cycles with no significant deactivation, thus offering a highly efficient and robust platform for the synthesis of organoboron compounds.

## 1. Introduction

**Scheme 1:**
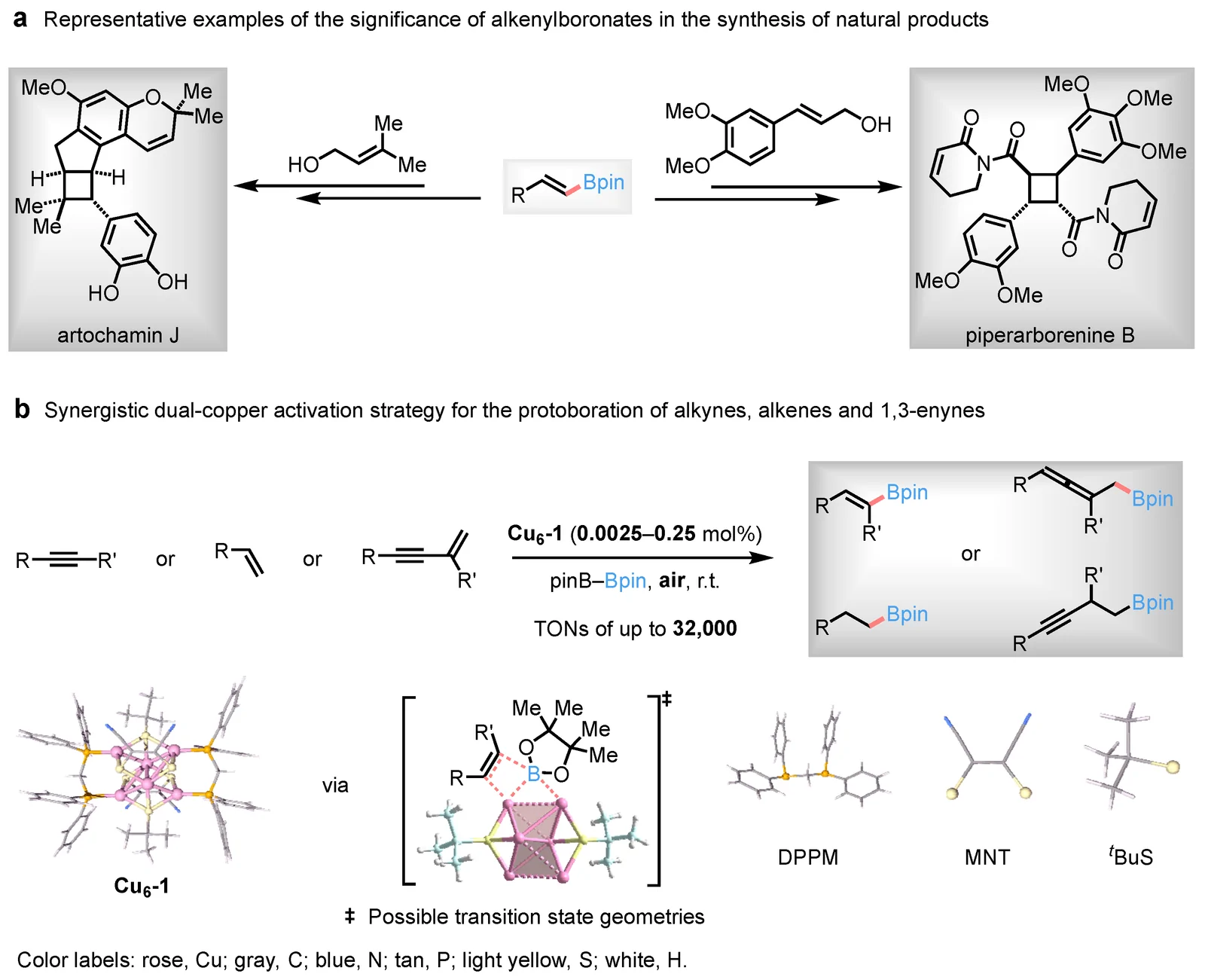
Copper-catalyzed protoboration reactions and the applications of alkenyl boronate products. (**a**) Representative examples of the significance of alkenyl boronates in the synthesis of natural products. (**b**) Synergistic dual-copper activation strategy for the protoboration of alkynes, alkenes and 1,3-enynes.

Organoboron compounds, especially alkenylboron reagents, have become key intermediates in organic synthesis [1,2,3,4,5,6], medicinal chemistry [7,8] (e.g., Dutogliptin [9] and Pummerer ketone [10]), organic semiconductors [11], and materials science [12] due to their excellent functional group tolerance, structural tunability, and low toxicity [13]. As versatile building blocks, alkenylboron reagents are not only widely used in key transformations of cross-coupling reactions such as Suzuki–Miyaura [14,15], but are also frequently employed in the total synthesis of complex natural products (Scheme 1a) [16] (e.g., Artochamin J and Piperarborenine B [17], Heliespirone A and Heliannuol E [18]) and the preparation of marketed drugs (e.g., Velcade and Nebivolol [19]). Among numerous synthetic strategies, the hydroboration (protoboration) of alkynes is one of the most attractive, efficient, and direct routes to access alkenyl boronates [20,21,22,23,24,25]. Despite the considerable progress achieved in transition-metal-catalyzed hydroboration [26,27,28] (both homogeneous and heterogeneous), many limitations persist: homogeneous systems usually require high catalyst loadings and are difficult to recover; heterogeneous catalysts suffer from ill-defined surface atomic structures, making it challenging to elucidate the catalytic mechanisms, which hinders the rational design and performance optimization of catalysts. Therefore, developing novel catalytic systems that combine high activity, excellent selectivity, well-defined structures, and stable recyclability has become an important challenge in this field.

In recent years, atomically precise metal nanoclusters, as an emerging nanomaterial, have attracted considerable attention in organic synthesis [29,30,31] due to their abundant active sites, high stability, high dispersibility, and good recyclability. Their high reactivity, selectivity, and efficiency mainly stem from the large specific surface area of nanocatalysts, unique size effects, tunable ligand frameworks, and well-defined core structures [30,32,33,34,35]. Based on these characteristics, nanometal catalysts have been successfully applied in a broad range of catalytic reactions [36,37,38,39,40,41,42,43,44,45]. However, in the hydroboration (protoboration) of alkynes, although copper-core nanoclusters such as **Au_1_Cu_14_** [46], **Cu_45_** [47], **Cu_4_** [48], and [**Cu–Cu_54_**] [49] have exhibited excellent catalytic activity, significant difficulties remain in the transformation of more challenging substrates such as alkenes and enynes.

As a “bridge” material connecting homogeneous and heterogeneous catalysis, metal nanoclusters feature precise atomic compositions and tunable surface ligand environments, which not only facilitate the elucidation of reaction mechanisms at the molecular level but also offer possibilities for further enhancing catalytic performance through precise structural modifications. Herein, we report a class of stable copper nanoclusters with exposed active sites, suitable for catalyzing the protoboration of C–C multiple bonds (Scheme 1b). We constructed active copper nanoclusters on a gram scale at low cost and found that the presence of Cu–Cu bonds plays a key role in broadening the substrate scope of the protoboration reaction (simultaneously compatible with alkynes, alkenes, and 1,3-enynes). In terms of catalytic performance, we achieved efficient catalysis with copper nanoclusters under low catalyst loading in air and in aqueous systems, and the nanoclusters showed almost no loss of activity after multiple cycles. Their excellent stability was further confirmed by UV–Vis, NMR, and ESI-MS characterizations.

## 2. Results and Discussion

**Figure 1:**
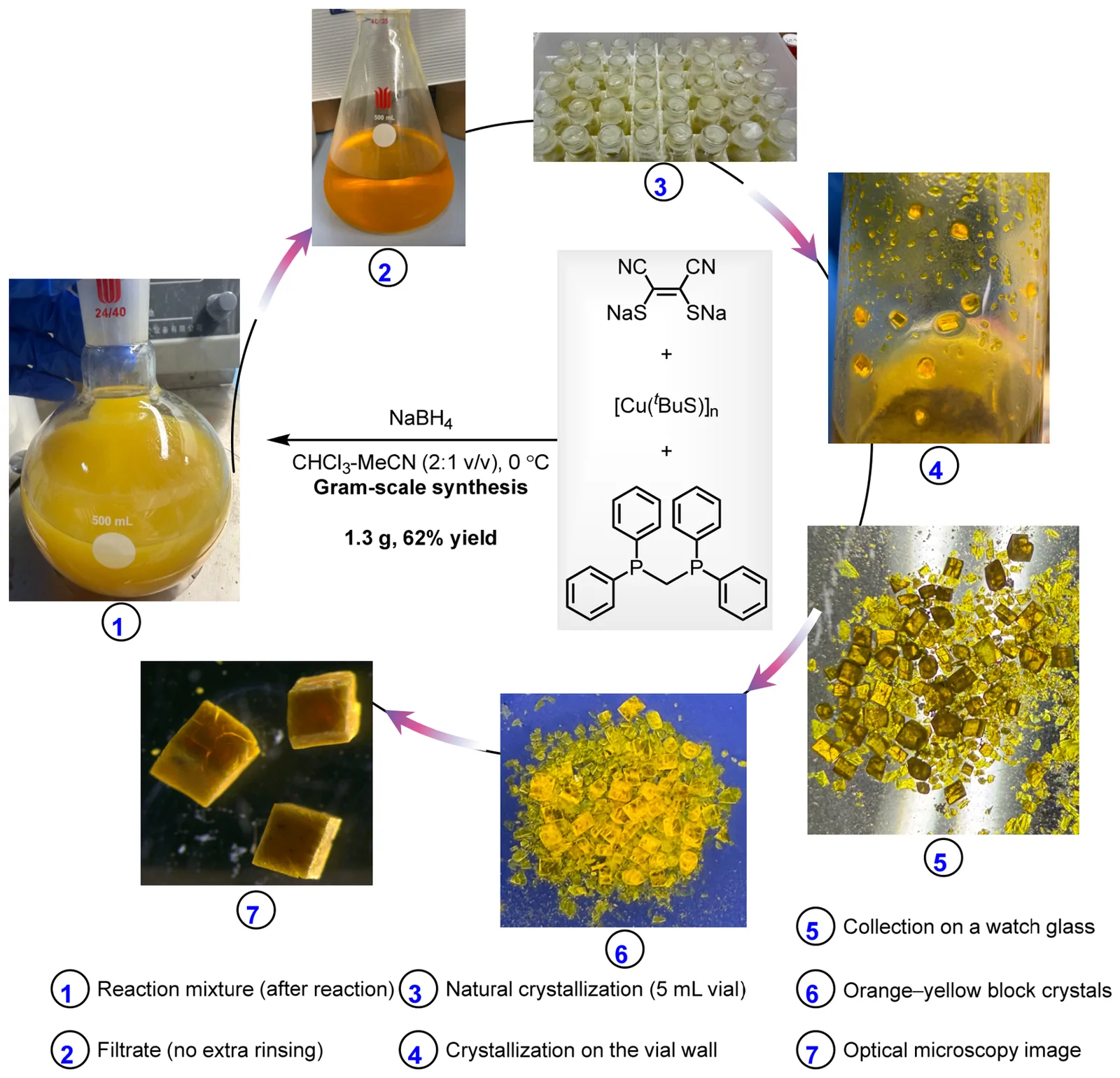
Gram-scale synthetic procedure for **Cu_6_-1**.

Gram-Scale Synthesis of the **Cu_6_-1** Nanocluster. In our previous work, we successfully synthesized two **Cu_6_** nanoclusters, each featuring a core with two exposed copper centers chelated by mnt ligands and applied them to the protosilylation of C–C unsaturated bonds [44]. Building on the atomically precise structure of **Cu_6_** nanoclusters as a foundation for scalable synthesis, we targeted **Cu_6_-1** and optimized the stoichiometry, crystallization, and enrichment procedures, thereby successfully achieving a facile gram-scale synthesis of **Cu_6_-1** nanocluster crystals (Figure 1).

To a 500 mL round-bottom flask under air were sequentially added [Cu(*^t^*BuS)]_n_ (1.2 g, 7.8 mmol), bis(diphenylphosphino)methane (dppm) (999.4 mg, 2.6 mmol), and CHCl_3_ (240 mL) at 0 °C. After 10 min, a solution of sodium maleonitriledithiolate (Na_2_mnt) (540 mg, 3.6 mmol) in MeCN (60 mL) was added. Following an additional 10 min, a solution of NaBH_4_ (240 mg, 6 mmol) in MeCN (60 mL) was introduced, and the mixture was stirred at 0 °C for 10 h. Upon completion, the mixture was filtered, and the filtrate was distributed into several 5 mL vials. The vials were allowed to stand in the dark at room temperature to permit slow solvent evaporation. After 48–96 h, abundant orange-yellow block crystals precipitated on the vial walls. The crystals were collected, washed with MeCN to remove impurities, and dried to afford **Cu_6_-1** as orange-yellow block crystals (1.3 g, 62%).

Initial Optimization Studies. This study commenced with an investigation of the protoboration of phenylacetylene with B_2_pin_2_ as a model reaction. Given that both **Cu_6_-1** and **Cu_6_-2** nanoclusters feature exposed active sites, we hypothesized that they could serve as potential high-performance catalysts for the protoboration reaction. Through a systematic screening of reaction parameters—including the copper catalyst, base, and solvent (Table 1)—we identified that the protoboration proceeded smoothly under air at room temperature in a mixed MeCN–MeOH solvent in the presence of either **Cu_6_-1** or **Cu_6_-2** and K_2_CO_3_, affording the desired product in 75% yield (**Cu_6_-1**) and 72% yield (**Cu_6_-2**), respectively (Table 1, entries 1 and 2). The influence of the catalyst was evaluated using a controlled variable approach. The results revealed that the two newly developed copper nanoclusters significantly outperformed other copper-based nanoclusters in promoting the protoboration reaction (entry 3). In contrast, conventional copper sources, including various Cu(I) and Cu(II) salts and their synthetic precursor [Cu(MeCN)_4_]BF_4_, proved to be far less effective than the cluster-based catalysts (entries 4–8). Subsequently, we assessed the effect of the base. The results indicated that weak inorganic bases (entries 9 and 10) were inferior to stronger ones (entries 11–13), with LiOH•H_2_O and LiO*^t^*Bu delivering particularly satisfactory yields. Notably, **Cu_6_-2** also achieved an excellent yield of 86% in the presence of LiO*^t^*Bu (entry 14). Further solvent-effect studies demonstrated that the participation of a protic solvent is crucial for this catalytic system (entries 15–18). To elucidate the reaction mechanism, dark-control experiments and radical trapping experiments were conducted. When the **Cu_6_-1**-catalyzed protoboration was performed in the dark, the yield remained nearly identical to that under natural light (entries 13 and 19), indicating that light irradiation is not a prerequisite for this organic transformation. Furthermore, the addition of the radical scavenger 2,2,6,6-tetramethylpiperidinyloxy (TEMPO) only marginally decreased the conversion to 92% (entry 20). A comprehensive analysis of these results rules out the possibility of a radical pathway (i.e., formation of boron radical species is unlikely [19,46,47]). Finally, critical blank control experiments confirmed the strict condition-dependence of this reaction: no reaction occurred upon removal of the copper catalyst or omission of the base (entries 21 and 22).

**Figure 2:**
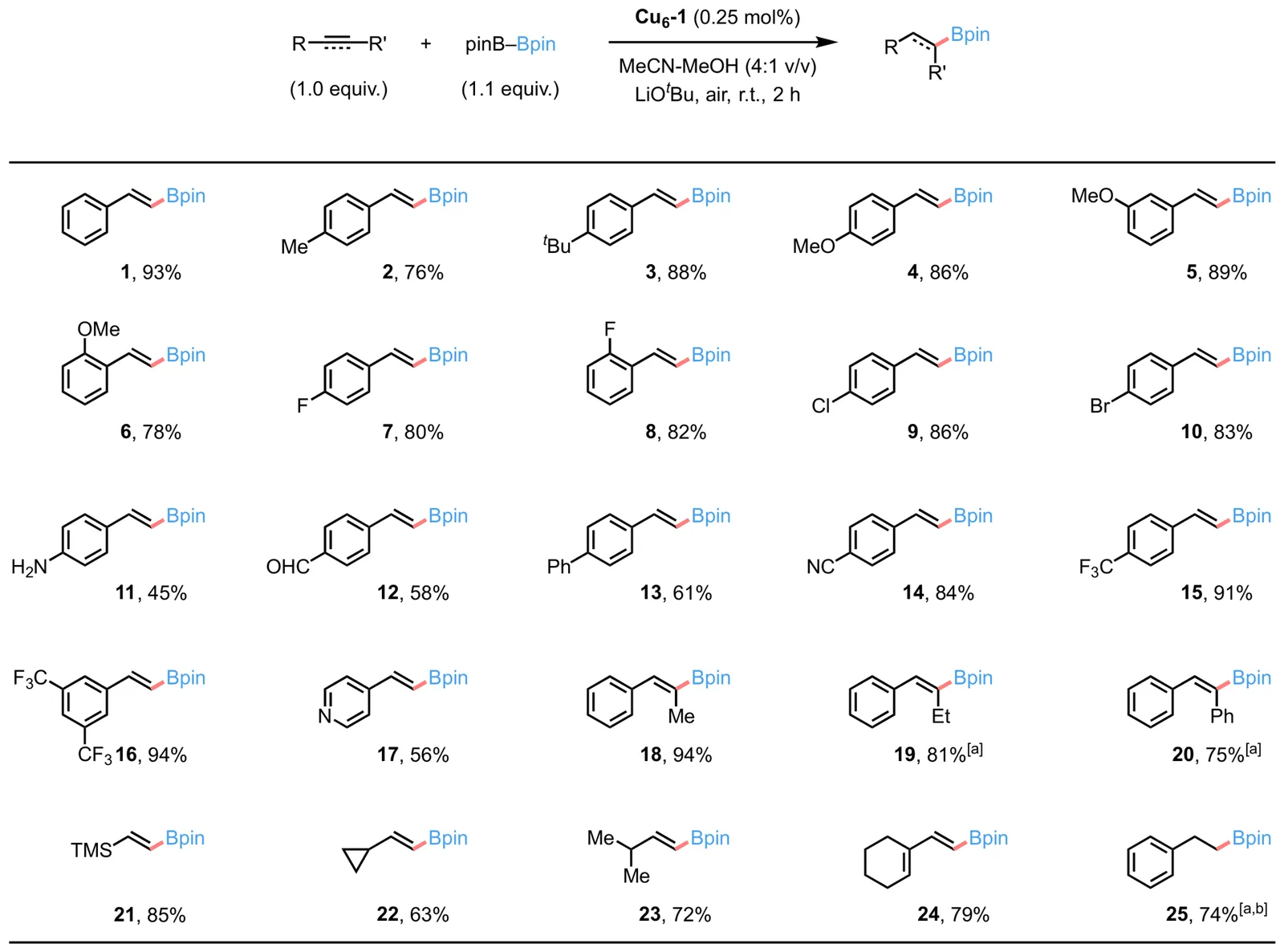
Substrate scope for protoboration with **Cu_6_-1**. Reaction conditions: alkyne or alkene (0.20 mmol, 1.0 equiv.), B_2_pin_2_ (1.1 equiv.), **Cu_6_-1** (0.25 mol%), and LiO*^t^*Bu (1.1 equiv.) in MeCN (1.6 mL) and MeOH (0.4 mL) under an air atmosphere at room temperature for 2 h. For each entry number (in bold), data are reported as isolated yields. Without additional notice, the regioisomeric ratios (r.r.) were found to exceed 20:1, as determined by ^1^H NMR analysis of the crude reaction mixture. ^[a]^ **Cu_6_-1** (0.5 mol%) was used. ^[b]^ 12 h.

Substrate Scope. After establishing the optimal reaction conditions, we further investigated the substrate scope of the **Cu_6_-1** cluster-catalyzed protoboration of C–C multiple bonds (Figure 2). The results demonstrate that a series of alkyne substrates bearing various substituted aryl groups underwent smooth reactions, affording the desired (*E*)-configured products with excellent regioselectivity (Figure 2, products **1–10** and **13–16**). Both electron-donating and electron-withdrawing functional groups at the ortho, meta, or para positions exhibited good functional group tolerance. It is worth noting that even aryl alkynes bearing sensitive functional groups (such as NH_2_ and CHO) and pyridine-based alkynes (products **11**, **12**, and **17**) proceeded smoothly. Although the yields decreased to some extent, these products serve as crucial intermediates in pharmaceutical synthesis and materials science. Furthermore, the **Cu_6_-1** catalytic system exhibited high compatibility with internal alkyne substrates (products **18–20**). However, an increase in the steric hindrance of the substituents on the internal alkynes significantly affected catalytic efficiency. As a special case, trimethylsilylacetylene, a commonly used organosilicon reagent, could be efficiently converted into (*E*)-trimethylsilyl alkenylboronate (product **21**) in a yield of up to 85%. In addition to aryl-terminal alkynes, alkyl-substituted terminal alkynes were also applicable to this protoboration system (products **22** and **23**), although the substrate containing a cyclopropyl group gave a relatively lower yield. Under standard conditions, the reaction also enabled the construction of 1,3-dienylboronates (product **24**) with high regio- and stereoselectivity. More importantly, the substrate scope could be further extended to alkene (product **25**), which was difficult to achieve with previous copper catalytic systems [48].

**Scheme 2:**
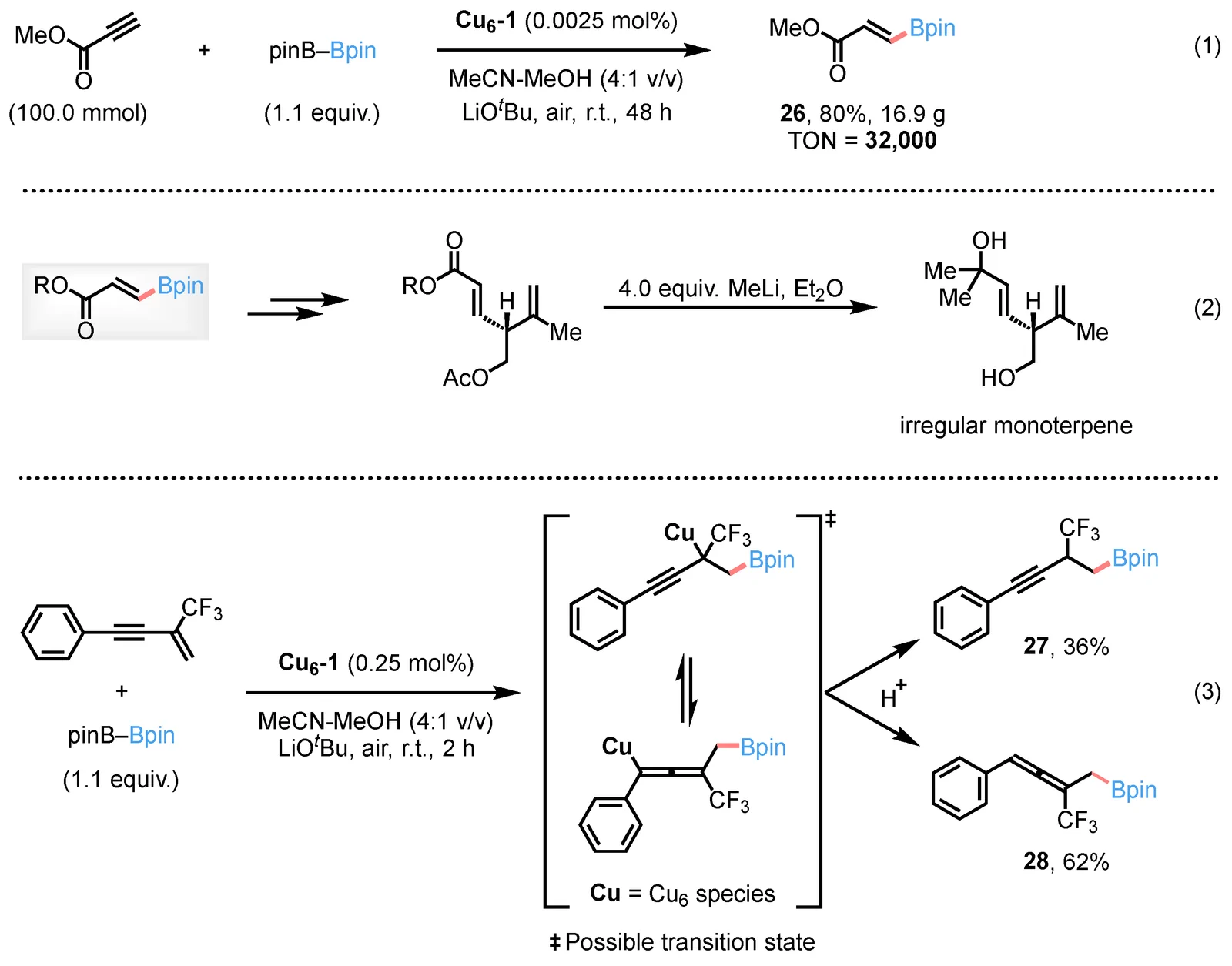
Gram-scale synthesis and the protoboration of 1,3-enyne.

Notably, under standard conditions, the gram-scale reaction of methyl propiolate proceeded smoothly even when the catalyst loading was reduced to 1/100, affording the α, β-unsaturated ester (Product **26**) with high regio- and stereoselectivity, and achieving a remarkably high turnover number (TON) of 32,000 (Scheme 2, (1)). As vital synthetic building blocks, α,β-unsaturated esters [18] can be transformed via highly efficient and selective conversion reactions to further synthesize an irregular monoterpene [50] derived from *Artemisia annua* L. (Scheme 2, (2)). More importantly, we extended the substrate scope to 2-trifluoromethyl enyne (Scheme 2, (3)). In this reaction, besides obtaining the target protoboration product **27** in 36% yield, the key intermediate for this transformation—the propargylcopper species—underwent 1,3-cupration to form an allenylcopper intermediate [51,52], which upon subsequent protonation afforded allene product **28** in 62% yield.

**Figure 3:**
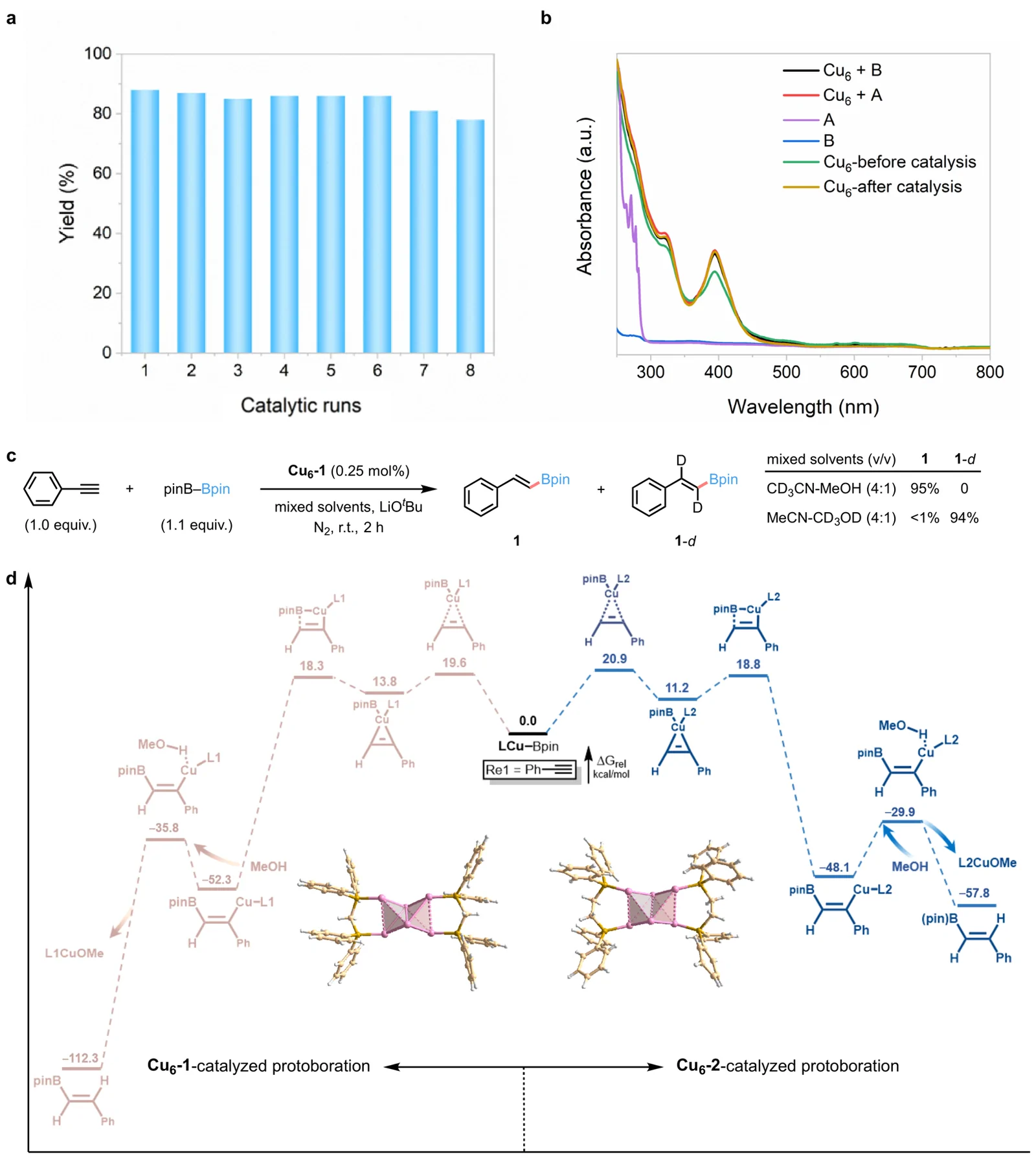
(**a**) Recycling experiments for cluster-catalyzed protoboration of phenylacetylene. (**b**) UV−Vis absorption spectra of **Cu_6_-1** before and after catalysis. A = phenylacetylene; B = B_2_pin_2_. (**c**) Deuterium labeling experiments. (**d**) DFT-calculated Gibbs free energy profile (Plausible mechanism for protoboration with **Cu_6_-1** or **Cu_6_-2**).

Recyclability and Mechanistic Investigations. The recyclability of the **Cu_6_-1** nanocluster catalyst was first systematically evaluated. As shown in Figure 3a, the catalyst retained excellent activity for the protoboration reaction after eight consecutive cycles without noticeable deactivation. Characterization of the samples before and after catalysis by UV–Vis absorption spectroscopy (Figure 3b), NMR (Figures S1 and S2), and ESI-MS (Figure S3) confirmed the preservation of the catalyst’s chemical composition, indicating that no decomposition occurred during the reaction. Notably, although distinct changes in UV–Vis absorbance intensity were observed when **Cu_6_-1** was mixed separately with phenylacetylene or B_2_pin_2_, the peak positions and shapes remained nearly perfectly superimposable. This not only confirms a discernible interaction between the substrates and the nanocluster but also demonstrates that such interactions do not compromise the structural integrity of the cluster, thereby providing a sound basis for its outstanding recyclability. Given the use of a MeCN-MeOH mixed-solvent strategy in the reaction system, isotopic labeling experiments with the corresponding deuterated solvents were conducted to trace the proton source in the catalytic system (Figure 3c). No deuterated product was detected when acetonitrile-d_3_ (CD_3_CN) was used in place of MeCN. In contrast, under a nitrogen atmosphere, substituting MeOH with methanol-*d*_4_ (CD_3_OD) afforded the deuterated product **1-*d*** in 94% yield, unequivocally establishing MeOH as the proton donor in this catalytic system. Furthermore, the formation of a doubly deuterated product implies that a cluster–alkynyl species may be generated under the reaction conditions and is subsequently protonated.

Based on the above experimental results and combined with DFT calculations, a plausible reaction mechanism is proposed in Figure 3d. When the **Cu_6_-1** nanocluster acts as the catalyst, with the assistance of base and MeOH, the alkyne coordinates to the exposed active site of the copper cluster to generate a cluster–Bpin intermediate. The alkyne molecule then binds laterally to this intermediate, triggering dissociation of one *^t^*BuS ligand and leading to the formation of a cyclopropenylcopper intermediate. In the subsequent Bpin migration step, a 1,2-insertion occurs, which is synergistically stabilized by two copper centers within a fused four-membered metallacycle and three-membered ring, resulting in an activation energy of only 18.3 kcal mol^−1^. This distinctive dual-copper-center migratory insertion mode enables the protoboration reaction to proceed efficiently at room temperature and successfully extends the substrate scope to C=C double bonds. The concerted insertion ultimately furnishes an alkenylcopper intermediate with the cluster unit and Bpin in a cis configuration. Protonolysis of this intermediate by MeOH generates the trans-alkene and concomitantly forms **L1CuOMe**. Finally, the initial **Cu_6_-1** cluster catalyst is regenerated via ligand dissociation.

## 3. Materials and Methods

### 3.1. General Information

Unless otherwise noted, all reagents and solvents were purchased from commercial suppliers and used without further purification or prepared according to literature procedures. All reactions were carried out in reaction tubes under an air atmosphere using solvents of analytical reagent (AR) grade. Column chromatography was performed using 100–200 or 200–300 mesh silica gel. Thin-layer chromatography (TLC) was performed on EM Reagents (EM Laboratories, Darmstadt, Germany) 0.25 mm silica gel 60-F plates.

Electrospray ionization time-of-flight (ESI-TOF) mass spectrometry data were recorded on a Waters Q-TOF mass spectrometer (Waters, Milford, MA, USA) using a Z-spray source. The ESI-TOF mass sample was prepared by dissolving the nanoclusters in dichloromethane (~0.5 mg mL^−1^). For positive-ion detection, the samples were directly infused into the chamber at 5 mL min^−1^. The source temperature was maintained at 70 °C, the spray voltage was 2.20 kV, and the cone voltage was adjusted to 60 V. High-resolution ESI mass measurements were performed on a Bruker Impact II high-resolution LC-QTOF mass spectrometer (Bruker, Billerica, MA, USA). Accurate masses from high-resolution mass spectra were reported for the molecular ion [M + Cs]^+^.

All UV−Vis absorption spectra were acquired in the 200−800 nm range using a Cary 3500 spectrophotometer (Agilent, Santa Clara, CA, USA).

^1^H NMR spectra were recorded on a Bruker 400 (400 MHz) or BRUKER 500 (500 MHz) spectrometer in CDCl_3_ (Bruker, Billerica, MA, USA). Chemical shifts were quoted in parts per million (ppm) referenced to 0.0 ppm for tetramethylsilane. Data are reported as follows: chemical shift, multiplicity (s = singlet, d = doublet, t = triplet, q = quartet, m = multiplet), coupling constants, *J*, were reported in Hertz (Hz). ^19^F NMR spectra were recorded on a BRUKER 400 (376 MHz) or BRUKER 500 (471 MHz) spectrometer with complete decoupling in CDCl_3_.

For geometry calculations, all computations were performed using the Gaussian 16 suite of programs (Revision C.01, Gaussian, Inc., Wallingford, CT, USA) [53]. For geometry optimizations, the X-ray crystal structures of copper nanoclusters **Cu_6_-1** and **Cu_6_-2** were adopted as starting geometries and fully optimized. The gradient-corrected B3LYP exchange-correlation functional [54], based on the generalized gradient approximation (GGA), was employed throughout. The SDD basis set [55] (Los Alamos effective core potential double-ζ) was used for Cu atoms, while the 6-31G(d) basis set was applied to C, H, P, S, and N atoms. Vibrational frequency calculations were carried out at the B3LYP/6-31G(d) level of theory to characterize all stationary points as minima (with the number of imaginary frequencies NIMAG = 0). Relative energies were corrected by vibrational zero-point energies (ZPE, unscaled). All energetic parameters were computed under standard conditions (298.15 K and 1 atm). Molecular representations were generated using GaussView (version 5; Semichem Inc., Shawnee Mission, KS, USA). The relative free energies (ΔG) discussed in the main text were calculated in the gas phase using the B3LYP/6-31G(d) method (with SDD for Cu atoms).

### 3.2. Synthesis of the [Cu(^t^BuS)]_n_ Precursor

A mixture of [Cu(MeCN)_4_]BF_4_ (3.1 g, 10.0 mmol) and tert-butyl mercaptan (*^t^*BuSH) (1.1 g, 12 mmol) was dissolved in acetonitrile (25 mL) under vigorous stirring at room temperature. Then, triethylamine was added to the reaction solution until a yellow powder precipitated, and the reaction continued for 12 h at room temperature. Finally, the yellow powder of [Cu(*^t^*BuS)]n was collected by filtration, washed with ethanol and ether, and dried under ambient conditions (1.4 g, 92% yield).

### 3.3. Cu_6_ Nanocluster-Catalyzed Protoboration of Alkynes and Alkenes

Alkyne (0.2 mmol), B_2_pin_2_ (55.9 mg, 0.22 mmol), **Cu_6_-1** (0.8 mg, 0.25 mol%), LiO*^t^*Bu (17.6 mg, 0.22 mmol), and MeCN-MeOH (*v*/*v* 1.6 mL/0.4 mL) were placed sequentially in a 10 mL glass tube with a magnetic stir bar. After the mixture was stirred at room temperature for 2 h, ethyl acetate (5 mL) was added to the crude mixture. The resulting solution was then filtered through a short plug of silica gel and washed three times with ethyl acetate (3 × 5 mL). The combined filtrates were concentrated under vacuum. The residue was purified by flash column chromatography on silica gel to afford the corresponding product.

*(E)-4,4,5,5-Tetramethyl-2-styryl-1,3,2-dioxaborolane* (**1**) [24]. The title compound was synthesized according to the General Procedure using phenylacetylene (20.4 mg, 0.2 mmol). The crude mixture was purified by flash column chromatography using hexane/ethyl acetate (10:1 *v*/*v*) as the eluent to give 42.8 mg (93% yield) of the title compound as a colorless oil.

^1^H NMR (CDCl_3_, 400 MHz): *δ* 7.52–7.46 (m, 2H), 7.40 (d, *J* = 18.5 Hz, 1H), 7.37–7.27 (m, 3H), 6.17 (d, *J* = 18.4 Hz, 1H), 1.32 (s, 12H).

*(E)-4,4,5,5-Tetramethyl-2-(4-methylstyryl)-1,3,2-dioxaborolane* (**2**) [24]. The title compound was synthesized according to the General Procedure using 1-ethynyl-4-methylbenzene (23.2 mg, 0.2 mmol). The crude mixture was purified by flash column chromatography using hexane/ethyl acetate (10:1 *v*/*v*) as the eluent to give 37.1 mg (76% yield) of the title compound as a colorless oil.

^1^H NMR (CDCl_3_, 400 MHz): *δ* 7.46–7.30 (m, 3H), 7.14 (d, *J* = 7.9 Hz, 2H), 6.11 (d, *J* = 18.5 Hz, 1H), 2.34 (s, 3H), 1.31 (s, 12H).

*(E)-2-(4-(tert-Butyl)styryl)-4,4,5,5-tetramethyl-1,3,2-dioxaborolane* (**3**) [46]. The title compound was synthesized according to the General Procedure using 1-(tert-butyl)-4-ethynylbenzene (31.6 mg, 0.2 mmol). The crude mixture was purified by flash column chromatography using hexane/ethyl acetate (10:1 *v*/*v*) as the eluent to give 50.4 mg (88% yield) of the title compound as a colorless solid.

^1^H NMR (CDCl_3_, 400 MHz): *δ* 7.44–7.35 (m, 5H), 6.12 (d, *J* = 18.4 Hz, 1H), 1.31 (s, 21H).

*(E)-2-(4-Methoxystyryl)-4,4,5,5-tetramethyl-1,3,2-dioxaborolane* (**4**) [24]. The title compound was synthesized according to the General Procedure using 1-ethynyl-4-methoxybenzene (26.4 mg, 0.2 mmol). The crude mixture was purified by flash column chromatography using hexane/ethyl acetate (10:1 *v*/*v*) as the eluent to give 44.7 mg (86% yield) of the title compound as a colorless oil.

^1^H NMR (CDCl_3_, 500 MHz): *δ* 7.44 (d, *J* = 8.3 Hz, 2H), 7.35 (d, *J* = 18.4 Hz, 1H), 6.87 (d, *J* = 8.3 Hz, 2H), 6.01 (dd, *J* = 18.4, 1.0 Hz, 1H), 3.81 (s, 3H), 1.31 (s, 12H).

*(E)-2-(3-Methoxystyryl)-4,4,5,5-tetramethyl-1,3,2-dioxaborolane* (**5**) [46]. The title compound was synthesized according to the General Procedure using 1-ethynyl-3-methoxybenzene (26.4 mg, 0.2 mmol). The crude mixture was purified by flash column chromatography using hexane/ethyl acetate (10:1 *v*/*v*) as the eluent to give 46.3 mg (89% yield) of the title compound as a colorless oil.

^1^H NMR (CDCl_3_, 500 MHz): *δ* 7.37 (d, *J* = 18.4 Hz, 1H), 7.28–7.21 (m, 1H), 7.08 (d, *J* = 7.7 Hz, 1H), 7.03 (s, 1H), 6.85 (dd, *J* = 8.7, 2.2 Hz, 1H), 6.16 (d, *J* = 18.4 Hz, 1H), 3.81 (s, 3H), 1.31 (s, 12H).

*(E)-2-(2-Methoxystyryl)-4,4,5,5-tetramethyl-1,3,2-dioxaborolane* (**6**) [46]. The title compound was synthesized according to the General Procedure using 1-ethynyl-2-methoxybenzene (26.4 mg, 0.2 mmol). The crude mixture was purified by flash column chromatography using hexane/ethyl acetate (10:1 *v*/*v*) as the eluent to give 40.6 mg (78% yield) of the title compound as a colorless oil.

^1^H NMR (CDCl_3_, 500 MHz): *δ* 7.77 (d, *J* = 18.6 Hz, 1H), 7.55 (d, *J* = 7.6 Hz, 1H), 7.34–7.20 (m, 1H), 6.93 (t, *J* = 7.5 Hz, 1H), 6.87 (d, *J* = 8.2 Hz, 1H), 6.18 (d, *J* = 18.6 Hz, 1H), 3.85 (s, 3H), 1.31 (s, 12H).

*(E)-2-(4-Fluorostyryl)-4,4,5,5-tetramethyl-1,3,2-dioxaborolane* (**7**) [24]. The title compound was synthesized according to the General Procedure using 1-ethynyl-4-fluorobenzene (24 mg, 0.2 mmol). The crude mixture was purified by flash column chromatography using hexane/ethyl acetate (10:1 *v*/*v*) as the eluent to give 39.7 mg (80% yield) of the title compound as a white solid.

^1^H NMR (CDCl_3_, 400 MHz): *δ* 7.52–7.41 (m, 2H), 7.35 (d, *J* = 18.4 Hz, 1H), 7.10–6.96 (m, 2H), 6.07 (d, *J* = 18.4 Hz, 1H), 1.31 (s, 12H).

^19^F NMR (376 MHz, CDCl_3_): *δ* −112.37–−112.40 (m, 1F).

*(E)-2-(2-Fluorostyryl)-4,4,5,5-tetramethyl-1,3,2-dioxaborolane* (**8**) [56]. The title compound was synthesized according to the General Procedure using 1-ethynyl-2-fluorobenzene (24 mg, 0.2 mmol). The crude mixture was purified by flash column chromatography using hexane/ethyl acetate (10:1 *v*/*v*) as the eluent to give 40.7 mg (82% yield) of the title compound as a colorless oil.

^1^H NMR (CDCl_3_, 400 MHz): *δ* 7.65–7.50 (m, 2H), 7.29–7.23 (m, 1H), 7.11 (t, *J* = 7.6 Hz, 1H), 7.06–7.01 (m, 1H), 6.24 (d, *J* = 18.6 Hz, 1H), 1.32 (s, 12H).

^19^F NMR (376 MHz, CDCl_3_): *δ* −112.17–−135.64 (m, 1F).

*(E)-2-(4-Chlorostyryl)-4,4,5,5-tetramethyl-1,3,2-dioxaborolane* (**9**) [24]. The title compound was synthesized according to the General Procedure using 1-chloro-4-ethynylbenzene (27.3 mg, 0.2 mmol). The crude mixture was purified by flash column chromatography using hexane/ethyl acetate (10:1 *v*/*v*) as the eluent to give 45.5 mg (86% yield) of the title compound as a white solid.

^1^H NMR (CDCl_3_, 400 MHz): *δ* 7.41 (d, *J* = 8.5 Hz, 2H), 7.37–7.27 (m, 3H), 6.13 (d, *J* = 18.5 Hz, 1H), 1.31 (s, 12H).

*(E)-2-(4-Bromostyryl)-4,4,5,5-tetramethyl-1,3,2-dioxaborolane* (**10**) [24]. The title compound was synthesized according to the General Procedure using 1-bromo-4-ethynylbenzene (36.2 mg, 0.2 mmol). The crude mixture was purified by flash column chromatography using hexane/ethyl acetate (10:1 *v*/*v*) as the eluent to give 51.3 mg (83% yield) of the title compound as a white solid.

^1^H NMR (CDCl_3_, 400 MHz): *δ* 7.52–7.42 (m, 2H), 7.37–7.27 (m, 3H), 6.15 (d, *J* = 18.4 Hz, 1H), 1.31 (s, 12H).

*(E)-4-(2-(4,4,5,5-Tetramethyl-1,3,2-dioxaborolan-2-yl)vinyl)aniline* (**11**) [57]. The title compound was synthesized according to the General Procedure using 4-ethynylaniline (23.4 mg, 0.2 mmol). The crude mixture was purified by flash column chromatography using hexane/ethyl acetate (10:1 *v*/*v*) as the eluent to give 22.1 mg (45% yield) of the title compound as a white solid.

^1^H NMR (CDCl_3_, 500 MHz): *δ* 8.10–6.83 (m, 3H), 6.55 (d, *J* = 8.4 Hz, 2H), 5.86 (d, *J* = 18.4 Hz, 1H), 3.72 (s, 2H), 1.23 (s, 12H).

*(E)-4-(2-(4,4,5,5-Tetramethyl-1,3,2-dioxaborolan-2-yl)vinyl)benzaldehyde* (**12**) [58]. The title compound was synthesized according to the General Procedure using 4-ethynylbenzaldehyde (26 mg, 0.2 mmol). The crude mixture was purified by flash column chromatography using hexane/ethyl acetate (10:1 *v*/*v*) as the eluent to give 29.9 mg (58% yield) of the title compound as a white solid.

^1^H NMR (CDCl_3_, 500 MHz): *δ* 10.00 (s, 1H), 7.86 (d, *J* = 7.9 Hz, 2H), 7.63 (d, *J* = 7.9 Hz, 2H), 7.43 (d, *J* = 18.4 Hz, 1H), 6.33 (d, *J* = 18.3 Hz, 1H), 1.33 (s, 12H).

*(E)-2-(2-([1,1’-Biphenyl]-4-yl)vinyl)-4,4,5,5-tetramethyl-1,3,2-dioxaborolane* (**13**) [59]. The title compound was synthesized according to the General Procedure using 4-ethynyl-1,1’-biphenyl (35.6 mg, 0.2 mmol). The crude mixture was purified by flash column chromatography using hexane/ethyl acetate (10:1 *v*/*v*) as the eluent to give 37.4 mg (61% yield) of the title compound as a white solid.

^1^H NMR (CDCl_3_, 500 MHz): *δ* 7.55–7.47 (m, 6H), 7.43–7.31 (m, 3H), 7.27 (t, *J* = 7.3 Hz, 1H), 6.14 (d, *J* = 18.4 Hz, 1H), 1.26 (s, 12H).

*(E)-4-(2-(4,4,5,5-Tetramethyl-1,3,2-dioxaborolan-2-yl)vinyl)benzonitrile* (**14**) [60]. The title compound was synthesized according to the General Procedure using 4-ethynylbenzonitrile (25.4 mg, 0.2 mmol). The crude mixture was purified by flash column chromatography using hexane/ethyl acetate (10:1 *v*/*v*) as the eluent to give 42.9 mg (84% yield) of the title compound as a white solid.

^1^H NMR (CDCl_3_, 400 MHz): *δ* 7.63 (d, *J* = 8.4 Hz, 2H), 7.55 (d, *J* = 8.4 Hz, 2H), 7.37 (d, *J* = 18.4 Hz, 1H), 6.28 (d, *J* = 18.5 Hz, 1H), 1.32 (s, 12H).

*(E)-4,4,5,5-Tetramethyl-2-(4-(trifluoromethyl)styryl)-1,3,2-dioxaborolane* (**15**) [24]. The title compound was synthesized according to the General Procedure using 1-ethynyl-4-(trifluoromethyl)benzene (34 mg, 0.2 mmol). The crude mixture was purified by flash column chromatography using hexane/ethyl acetate (10:1 *v*/*v*) as the eluent to give 54.3 mg (91% yield) of the title compound as a white solid.

^1^H NMR (CDCl_3_, 400 MHz): *δ* 7.68–7.51 (m, 4H), 7.40 (d, *J* = 18.4 Hz, 1H), 6.26 (d, *J* = 18.5 Hz, 1H), 1.32 (s, 12H).

^19^F NMR (376 MHz, CDCl_3_): *δ* -62.62 (s, 3F).

*(E)-2-(3,5-Bis(trifluoromethyl)styryl)-4,4,5,5-tetramethyl-1,3,2-dioxaborolane* (**16**) [61]. The title compound was synthesized according to the General Procedure using 1-ethynyl-3,5-bis(trifluoromethyl)benzene (47.6 mg, 0.2 mmol). The crude mixture was purified by flash column chromatography using hexane/ethyl acetate (10:1 *v*/*v*) as the eluent to give 68.8 mg (94% yield) of the title compound as a colorless oil.

^1^H NMR (CDCl_3_, 400 MHz): *δ* 7.88 (s, 2H), 7.78 (s, 1H), 7.42 (d, *J* = 18.4 Hz, 1H), 6.32 (d, *J* = 18.4 Hz, 1H), 1.32 (s, 12H).

^19^F NMR (376 MHz, CDCl_3_): *δ* −63.09 (s, 6F).

*(E)-4-(2-(4,4,5,5-Tetramethyl-1,3,2-dioxaborolan-2-yl)vinyl)pyridine* (**17**) [62]. The title compound was synthesized according to the General Procedure using 4-ethynylpyridine (20.6 mg, 0.2 mmol). The crude mixture was purified by flash column chromatography using hexane/ethyl acetate (10:1 *v*/*v*) as the eluent to give 25.9 mg (56% yield) of the title compound as a white solid.

^1^H NMR (CDCl_3_, 400 MHz): *δ* 8.59 (d, *J* = 6.2 Hz, 2H), 7.36–7.24 (m, 3H), 6.38 (d, *J* = 18.4 Hz, 1H), 1.32 (s, 12H).

*(E)-4,4,5,5-Tetramethyl-2-(1-phenylprop-1-en-2-yl)-1,3,2-dioxaborolane* (**18**) [24]. The title compound was synthesized according to the General Procedure using prop-1-yn-1-ylbenzene (23.2 mg, 0.2 mmol). The crude mixture was purified by flash column chromatography using hexane/ethyl acetate (10:1 *v*/*v*) as the eluent to give 45.9 mg (94% yield) of the title compound as a colorless oil.

^1^H NMR (CDCl_3_, 400 MHz): *δ* 7.46–7.30 (m, 4H), 7.29–7.16 (m, 2H), 1.99 (s, 3H), 1.31 (s, 12H).

*(E)-4,4,5,5-Tetramethyl-2-(1-phenylbut-1-en-2-yl)-1,3,2-dioxaborolane* (**19**) [24]. The title compound was synthesized according to the General Procedure using **Cu_6_-1** (1.6 mg, 0.5 mol%) and but-1-yn-1-ylbenzene (26 mg, 0.2 mmol). The crude mixture was purified by flash column chromatography using hexane/ethyl acetate (10:1 *v*/*v*) as the eluent to give 41.8 mg (81% yield) of the title compound as a colorless oil.

^1^H NMR (CDCl_3_, 400 MHz): *δ* 7.36–7.31 (m, 4H), 7.28–7.17 (m, 2H), 2.39 (q, *J* = 7.4 Hz, 2H), 1.31 (s, 12H), 1.10 (t, *J* = 7.5 Hz, 3H).

*(E)-2-(1,2-Diphenylvinyl)-4,4,5,5-tetramethyl-1,3,2-dioxaborolane* (**20**) [24]. The title compound was synthesized according to the General Procedure using **Cu_6_-1** (1.6 mg, 0.5 mol%) and 1,2-diphenylethyne (35.6 mg, 0.2 mmol). The crude mixture was purified by flash column chromatography using hexane/ethyl acetate (10:1 *v*/*v*) as the eluent to give 45.9 mg (75% yield) of the title compound as a white solid.

^1^H NMR (CDCl_3_, 500 MHz): *δ* 7.36 (s, 1H), 7.29–7.23 (m, 2H), 7.23–7.18 (m, 1H), 7.16 (d, *J* = 8.1 Hz, 2H), 7.12–7.10 (m, 3H), 7.07–7.04 (m, 2H), 1.32 (s, 12H).

*(E)-Trimethyl(2-(4,4,5,5-tetramethyl-1,3,2-dioxaborolan-2-yl)vinyl)silane* (**21**) [63]. The title compound was synthesized according to the General Procedure using ethynyltrimethylsilane (19.6 mg, 0.2 mmol). The crude mixture was purified by flash column chromatography using hexane/ethyl acetate (10:1 *v*/*v*) as the eluent to give 38.5 mg (85% yield) of the title compound as a colorless oil.

^1^H NMR (CDCl_3_, 400 MHz): *δ* 7.10 (d, *J* = 21.8 Hz, 1H), 6.22 (d, *J* = 21.8 Hz, 1H), 1.26 (s, 12H), 0.06 (s, 9H).

*(E)-2-(2-Cyclopropylvinyl)-4,4,5,5-tetramethyl-1,3,2-dioxaborolane* (**22**) [47]. The title compound was synthesized according to the General Procedure using ethynylcyclopropane (13.2 mg, 0.2 mmol). The crude mixture was purified by flash column chromatography using hexane/ethyl acetate (10:1 *v*/*v*) as the eluent to give 24.5 mg (63% yield) of the title compound as a colorless oil.

^1^H NMR (CDCl_3_, 400 MHz): δ 6.06 (dd, *J* = 17.8, 9.3 Hz, 1H), 5.47 (d, *J* = 17.8 Hz, 1H), 1.56–1.44 (m, 1H), 1.24 (s, 12H), 0.83–0.75 (m, 2H), 0.55–0.49 (m, 2H).

*(E)-4,4,5,5-Tetramethyl-2-(3-methylbut-1-en-1-yl)-1,3,2-dioxaborolane* (**23**) [64]. The title compound was synthesized according to the General Procedure using 3-methylbut-1-yne (13.6 mg, 0.2 mmol). The crude mixture was purified by flash column chromatography using hexane/ethyl acetate (10:1 *v*/*v*) as the eluent to give 28.2 mg (72% yield) of the title compound as a colorless oil.

^1^H NMR (CDCl_3_, 400 MHz): δ 6.61 (dd, *J* = 18.1, 6.1 Hz, 1H), 5.37 (dd, *J* = 18.1, 1.5 Hz, 1H), 2.44–2.23 (m, 1H), 1.26 (s, 12H), 1.00 (d, *J* = 6.8 Hz, 6H).

*(E)-2-(2-(Cyclohex-1-en-1-yl)vinyl)-4,4,5,5-tetramethyl-1,3,2-dioxaborolane* (**24**) [49]. The title compound was synthesized according to the General Procedure using 1-ethynylcyclohex-1-ene (21.2 mg, 0.2 mmol). The crude mixture was purified by flash column chromatography using hexane/ethyl acetate (10:1 *v*/*v*) as the eluent to give 37.0 mg (79% yield) of the title compound as a pale yellow oil.

^1^H NMR (CDCl_3_, 400 MHz): δ 7.02 (d, *J* = 18.2 Hz, 1H), 5.96 (t, *J* = 4.0 Hz, 1H), 5.42 (d, *J* = 18.2 Hz, 1H), 2.17–2.10 (m, 4H), 1.69–1.62 (m, 2H), 1.62–1.55 (m, 2H), 1.27 (s, 12H).

*4,4,5,5-Tetramethyl-2-phenethyl-1,3,2-dioxaborolane* (**25**) [19]. The title compound was synthesized according to the General Procedure using styrene (20.8 mg, 0.2 mmol). The crude mixture was purified by flash column chromatography using hexane/ethyl acetate (10:1 *v*/*v*) as the eluent to give 34.4 mg (74% yield) of the title compound as a colorless oil.

^1^H NMR (CDCl_3_, 400 MHz): δ 7.31–7.22 (m, 4H), 7.20–7.15 (m, 1H), 2.78 (t, *J* = 8.4 Hz, 2H), 1.25 (s, 12H), 1.17 (t, *J* = 8.4 Hz, 2H).

### 3.4. Optimal Conditions for High Turnover Experiments

*Methyl (E)-3-(4,4,5,5-tetramethyl-1,3,2-dioxaborolan-2-yl)acrylate* (**26**) [57]. To a 2000 mL round-bottom flask under an air atmosphere were added **Cu_6_-1** (4.0 mg, 0.0025 mol%, S/C = 40,000), methyl propiolate (8.4 g, 100 mmol), B_2_pin_2_ (27.9 g, 110 mmol), LiO*^t^*Bu (8.8 g, 110 mmol), and MeCN-MeOH (800 mL/200 mL) sequentially. The reaction mixture was stirred at room temperature for 48 h. Then ethyl acetate (500 mL) was added to the crude mixture, and the resulting solution was filtered through a short plug of silica gel, and the plug was washed with ethyl acetate (3 × 500 mL). The combined filtrates were dried over anhydrous Na_2_SO_4_ and concentrated under vacuum. The residue was purified by flash column chromatography using hexane/ethyl acetate (5:1 *v*/*v*) as the eluent to give 16.9 g (80.0 mmol, 80% yield, TON = 32,000) of the title compound as a colorless solid.

^1^H NMR (CDCl_3_, 400 MHz): δ 6.77 (d, *J* = 18.2 Hz, 1H), 6.62 (d, *J* = 18.2 Hz, 1H), 3.75 (s, 3H), 1.27 (s, 12H).

### 3.5. Cu_6_ Nanocluster-Catalyzed Protoboration of 1,3-Enyne

(3-(Trifluoromethyl)but-3-en-1-yn-1-yl)benzene (39.2 mg, 0.2 mmol), B_2_pin_2_ (55.9 mg, 0.22 mmol), **Cu_6_-1** (0.8 mg, 0.25 mol%), LiO*^t^*Bu (17.6 mg, 0.22 mmol) and MeCN/MeOH (*v*/*v* 1.6 mL/0.4 mL) were placed sequentially in a 10 mL glass tube with a magnetic stir bar. After the mixture was stirred at room temperature for 2 h, ethyl acetate (5 mL) was added to the crude mixture. The resulting solution was then filtered through a short plug of silica gel and washed three times with ethyl acetate (3 × 5 mL). The combined filtrates were concentrated under vacuum. The crude mixture was purified by flash column chromatography using hexane/ethyl acetate (10:1 *v*/*v*) as the eluent to give 23.3 mg (36% yield) of compound **27** as a colorless oil and 40.2 mg (62% yield) of compound **28** as a colorless oil.

*4,4,5,5-Tetramethyl-2-(4-phenyl-2-(trifluoromethyl)but-3-yn-1-yl)-1,3,2-dioxaborolane* (**27**) [52].

^1^H NMR (CDCl_3_, 500 MHz): *δ* 7.36–7.32 (m, 2H), 7.26–7.19 (m, 3H), 3.47 (h, *J* = 7.8 Hz, 1H), 1.28 (d, *J* = 7.8 Hz, 2H), 1.19 (s, 12H).

^19^F NMR (471 MHz, CDCl_3_): δ −73.0 (d, *J* = 7.8 Hz, 3F).

*4,4,5,5-Tetramethyl-2-(4-phenyl-2-(trifluoromethyl)buta-2,3-dien-1-yl)-1,3,2-dioxaborolane* (**28**) [52].

^1^H NMR (CDCl_3_, 500 MHz): *δ* 7.40–7.35 (m, 2H), 7.35–7.29 (m, 3H), 6.53 (q, *J* = 3.1 Hz, 1H), 1.85 (ddd, *J* = 24.4, 16.3, 2.5 Hz, 2H), 1.22 (s, 6H), 1.18 (s, 6H).

^19^F NMR (471 MHz, CDCl_3_): δ −65.2 (d, *J* = 3.6 Hz).

### 3.6. Recycling Experiments

**Cu_6_-1**-catalyzed protoboration of phenylacetylene (Table S1). In the recycling experiments, phenylacetylene (20.4 mg, 0.2 mmol), B_2_pin_2_ (55.9 mg, 0.22 mmol), **Cu_6_-1** (1.6 mg, 0.5 mol%), K_2_CO_3_ (30.4 mg, 0.22 mmol), and MeCN-H_2_O (*v*/*v* 1.6 mL/0.4 mL) were placed sequentially in a 10 mL glass tube with a magnetic stir bar. After the mixture was stirred at room temperature for 20 min, **Cu_6_-1** was recovered by centrifugation and washed three times with MeCN-H_2_O (*v*/*v* 4 mL/1 mL). The resulting powder **Cu_6_-1** was used in the next cycle. Then the resulting solution was filtered through a short plug of silica gel and washed three times with ethyl acetate (3 × 5 mL). The yield was determined by gas chromatography using dodecane as an internal standard.

### 3.7. Deuterium Labeling Experiments

Phenylacetylene (20.4 mg, 0.2 mmol), B_2_pin_2_ (55.9 mg, 0.22 mmol), **Cu_6_-1** (0.8 mg, 0.25 mol%), LiO*^t^*Bu (17.6 mg, 0.22 mmol) and CD_3_CN-MeOH (*v*/*v* 1.6 mL/0.4 mL) were placed sequentially in a 10 mL glass tube with a magnetic stir bar. After the mixture was stirred at room temperature for 2 h, ethyl acetate (5 mL) was added to the crude mixture. Then the resulting solution was filtered through a short plug of silica gel and washed three times with ethyl acetate (3 × 5 mL). The combined filtrates were concentrated under vacuum. Yield was determined by ^1^H NMR of the crude product using mesitylene as an internal standard.

Phenylacetylene (20.4 mg, 0.2 mmol), B_2_pin_2_ (55.9 mg, 0.22 mmol), **Cu_6_-1** (0.8 mg, 0.25 mol%), LiO*^t^*Bu (17.6 mg, 0.22 mmol) and MeCN-CD_3_OD (*v*/*v* 1.6 mL/0.4 mL) were placed sequentially in a 10 mL glass tube with a magnetic stir bar. After the mixture was stirred at room temperature for 2 h, ethyl acetate (5 mL) was added to the crude mixture. Then the resulting solution was filtered through a short plug of silica gel and washed three times with ethyl acetate (3 × 5 mL). The combined filtrates were concentrated under vacuum. Yield was determined by ^1^H NMR of the crude product using mesitylene as an internal standard.

*(E)-4,4,5,5-Tetramethyl-2-(2-phenylvinyl-1,2-d_2_)-1,3,2-dioxaborolane* (**1**-*d*) [57]. ^1^H NMR (CDCl_3_, 400 MHz): δ 7.52–7.46 (m, 2H), 7.37–7.28 (m, 3H), 1.32 (s, 12H).

## 4. Conclusions

In this study, we successfully synthesized and characterized a class of copper nanoclusters (**Cu_6_-1**) with an atomically precise structure, and systematically investigated their catalytic performance in the protoboration of C–C unsaturated bonds. As a structurally well-defined and recyclable heterogeneous catalyst, **Cu_6_-1** serves as a powerful tool for the efficient construction of organoboron compounds and lays a solid theoretical and experimental foundation for the rational design of next-generation high-performance cluster-based catalysts.

## Figures and Tables

**Table 1:** Copper-nanocluster-catalyzed protoboration of phenylacetylene: effect of reaction parameters ^[a]^.

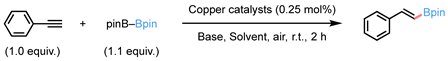				
**Entry**	**Copper Catalysts**	**Base**	**Solvent**	**Yield (%)**
1	**Cu_6_-1**	K_2_CO_3_	MeCN-MeOH	75
2	**Cu_6_-2**	K_2_CO_3_	MeCN-MeOH	72
3	**Cu_40_**	K_2_CO_3_	MeCN-MeOH	42
4 ^[b]^	[Cu(MeCN)_4]_BF_4_	K_2_CO_3_	MeCN-MeOH	20
5 ^[b]^	CuCl	K_2_CO_3_	MeCN-MeOH	21
6 ^[b]^	CuI	K_2_CO_3_	MeCN-MeOH	17
7 ^[b]^	Cu_2_O	K_2_CO_3_	MeCN-MeOH	22
8 ^[b]^	CuSO_4_	K_2_CO_3_	MeCN-MeOH	49
9	**Cu_6_-1**	Na_2_CO_3_/Cs_2_CO_3_	MeCN-MeOH	72/59
10	**Cu_6_-1**	KOAc/CsOAc	MeCN-MeOH	43/52
11	**Cu_6_-1**	LiOH•H_2_O	MeCN-MeOH	89
12	**Cu_6_-1**	KO*^t^*Bu/NaO*^t^*Bu	MeCN-MeOH	83/80
13	**Cu_6_-1**	LiO*^t^*Bu	MeCN-MeOH	95 (93) ^[c]^
14	**Cu_6_-2**	LiO*^t^*Bu	MeCN-MeOH	86
15	**Cu_6_-1**	LiO*^t^*Bu	MeCN	43
16	**Cu_6_-1**	LiO*^t^*Bu	MeOH	78
17	**Cu_6_-1**	LiO*^t^*Bu	THF	31
18	**Cu_6_-1**	LiO*^t^*Bu	EA	37
19 ^[d]^	**Cu_6_-1**	LiO*^t^*Bu	MeCN-MeOH	93
20 ^[e]^	**Cu_6_-1**	LiO*^t^*Bu	MeCN-MeOH	92
21	no catalyst	LiO*^t^*Bu	MeCN-MeOH	n.r.
22	**Cu_6_-1**	no base	MeCN-MeOH	trace

^[a]^ Reaction conditions: phenylacetylene (0.20 mmol, 1.0 equiv.), B_2_pin_2_ (1.1 equiv.), copper catalyst (0.25 mol%), and base (1.1 equiv.) in solvent (2.0 mL) under an air atmosphere at room temperature for 2 h. Yield was determined by ^1^H NMR of the crude product using mesitylene as an internal standard. ^[b]^ Copper catalyst (2.0 mol%) was used. ^[c]^ The yield refers to the isolated yield. ^[d]^ The reaction was conducted in the dark. ^[e]^ TEMPO (1.1 equiv.) was used as an additive. **Cu_40_** = [Cu_40_H_17_(2,4-DMBT)_24_](PPh_4_) [43]; MeCN-MeOH (4:1 *v*/*v*).

## Data Availability

The data underlying this study are available in the published article and its Supplementary Information (characterization data for all compounds).

## Supplementary Materials

The following supporting information can be downloaded at: https://www.mdpi.com/article/10.3390/molecules31172956/s1, Section S1: Table and Figures: Table S1: Recycling experiments for **Cu_6_-1**-catalyzed protoboration of phenylacetylene; Figure S1: ^1^H NMR of **Cu_6_-1** before and after recycling test (8 catalytic runs); Figure S2: ^31^P NMR of **Cu_6_-1** before and after recycling test (8 catalytic runs); Figure S3: HRMS of **Cu_6_-1** before and after recycling test (8 catalytic runs); Section S2: Supplementary NMR Spectra.

## Abbreviations

The following abbreviations are used in this manuscript:

DPPM	Bis(diphenylphosphino)methane
ESI-TOF	Electrospray ionization time-of-flight
GGA	Generalized gradient approximation
TON	Turnover number
TEMPO	2,2,6,6-Tetramethylpiperidinyloxy
TLC	Thin-layer chromatography
ZPE	Zero-point energies
