# Supplementary material for: Copper Nanoclusters with Exposed Active Sites for Highly Efficient Protoboration of C–C Multiple Bonds
Source: Molecules. 2026 Aug 24;31(17):2956. doi: 10.3390/molecules31172956 (PMC13567063; doi:10.3390/molecules31172956)
Supplement: Supplementary file 1 [file molecules-31-02956-s001.zip › molecules-4512995-supplementary.pdf]

Supplementary Information

Copper Nanoclusters with Exposed Active Sites for Highly Efficient Protoboration of C–C Multiple Bonds

Shaoke Zhang [1,2*#] and Songwei Wen [2*#]

[1] Advanced Institute for Ocean Research (AIOR), Southern University of Science and Technology (SUS-Tech), Shenzhen 518055, P. R. China
[2] Department of Chemistry, Southern University of Science and Technology (SUSTech), Shenzhen 518055, P. R. China
* Correspondence: zhangsk@sustech.edu.cn (S.Z.); wensw@sustech.edu.cn (S.W.); Tel.: +86-15071411923 (S.W.)
[#] S.Z. and S.W. contributed equally to this work

Supplementary Section S1. Tables and Figures

Table S1. Recycling Experiments for **Cu_6-1**-Catalyzed Protoboration of Phenylacetylene

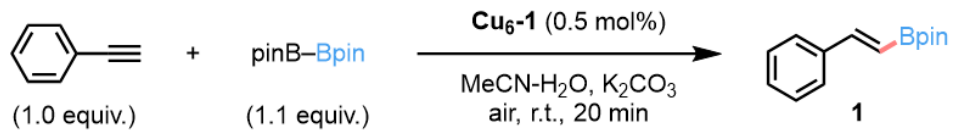

Cycle	Yield
1	88%
2	87%
3	85%
4	86%
5	86%
6	86%
7	81%
8	73%

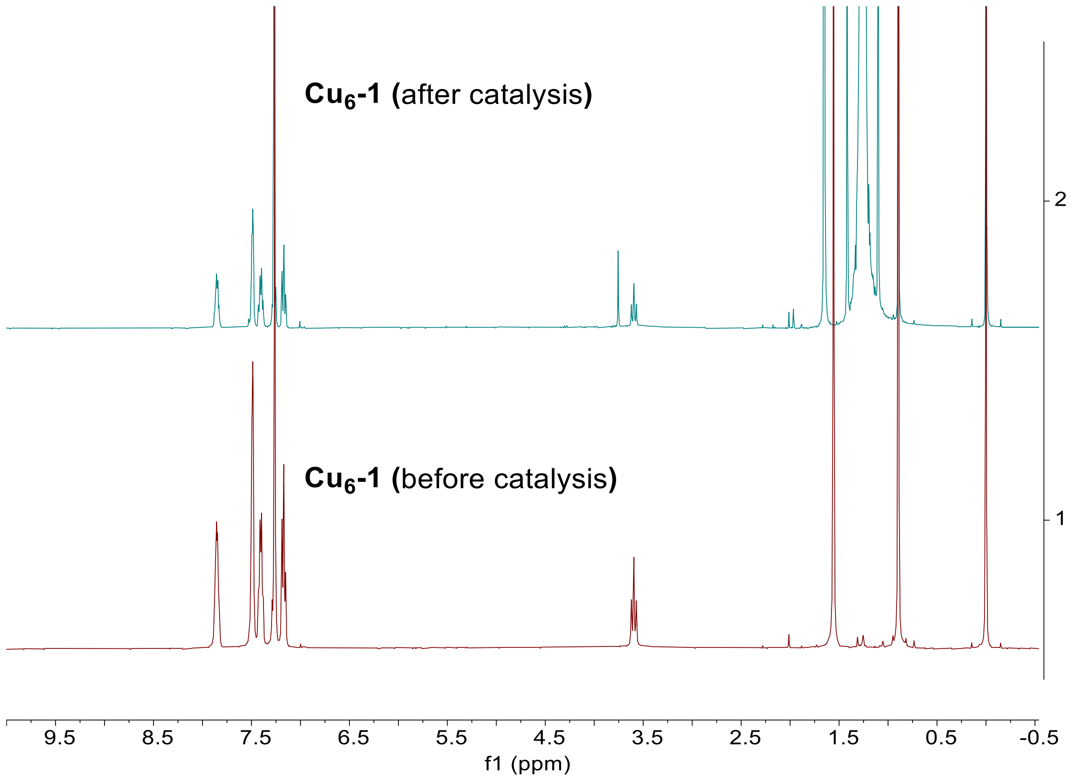

Figure S1. ^{1}H NMR of **Cu₆-1** before and after recycling test (8 catalytic runs).

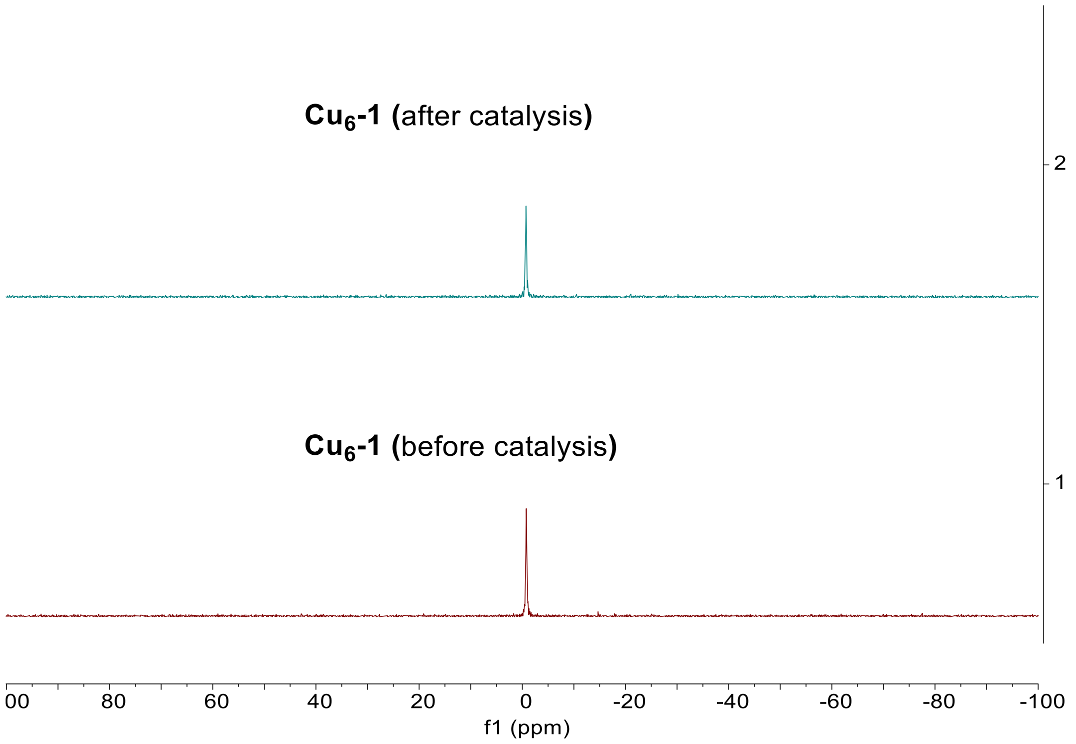

Figure S2. ^{31}P NMR of **Cu₆-1** before and after recycling test (8 catalytic runs).

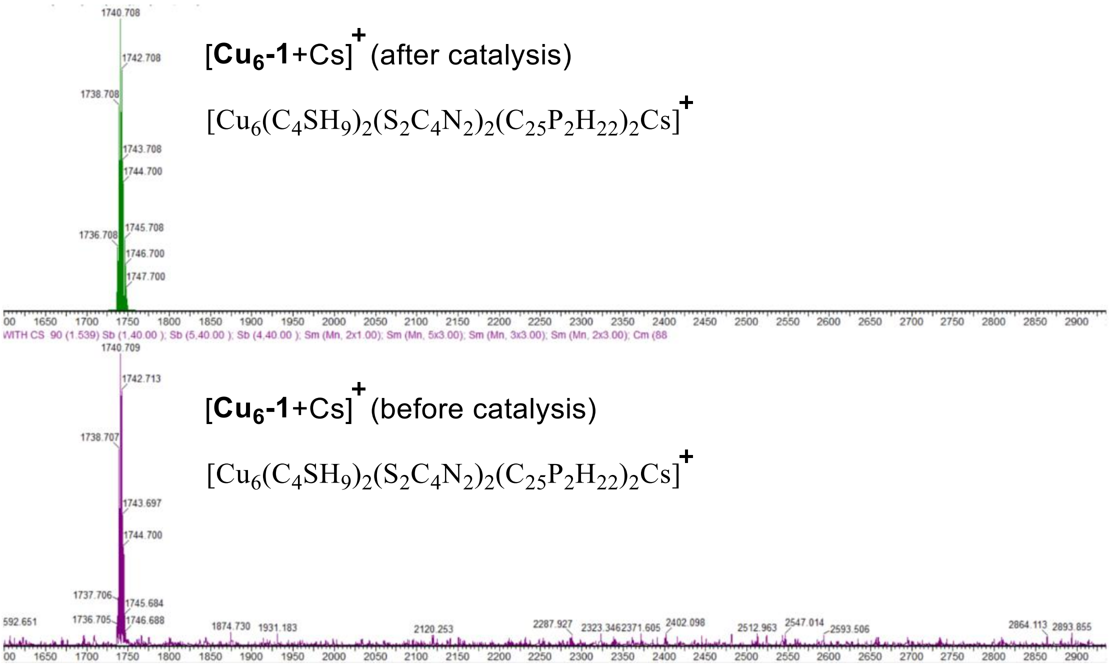

Figure S3. HRMS of **Cu₆-1** before and after recycling test (8 catalytic runs).

Section S2. Supplementary NMR Spectra

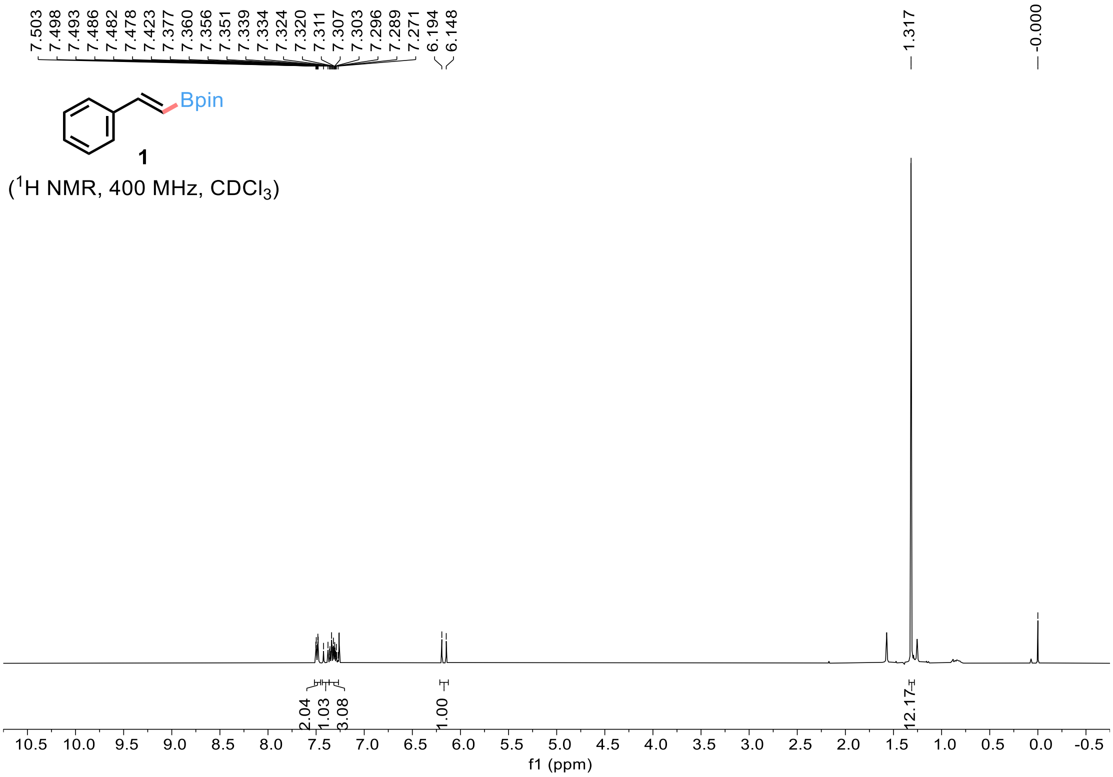

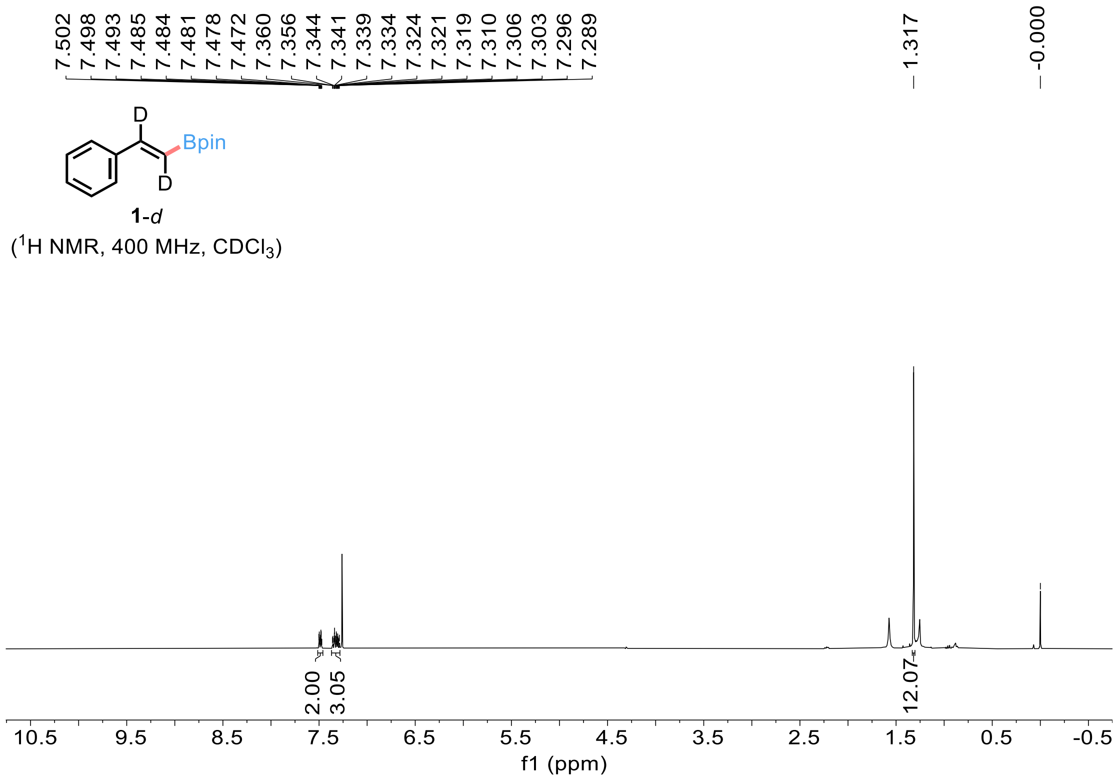
7.502
7.498
7.493
7.485
7.484
7.481
7.478
7.472
7.360
7.356
7.344
7.341
7.339
7.334
7.324
7.321
7.319
7.310
7.306
7.303
7.296
7.289
1.317
-0.000
D
Bpin
D
1-*d*
(¹H NMR, 400 MHz, CDCl3)
2.00
3.05
12.07
f1 (ppm)

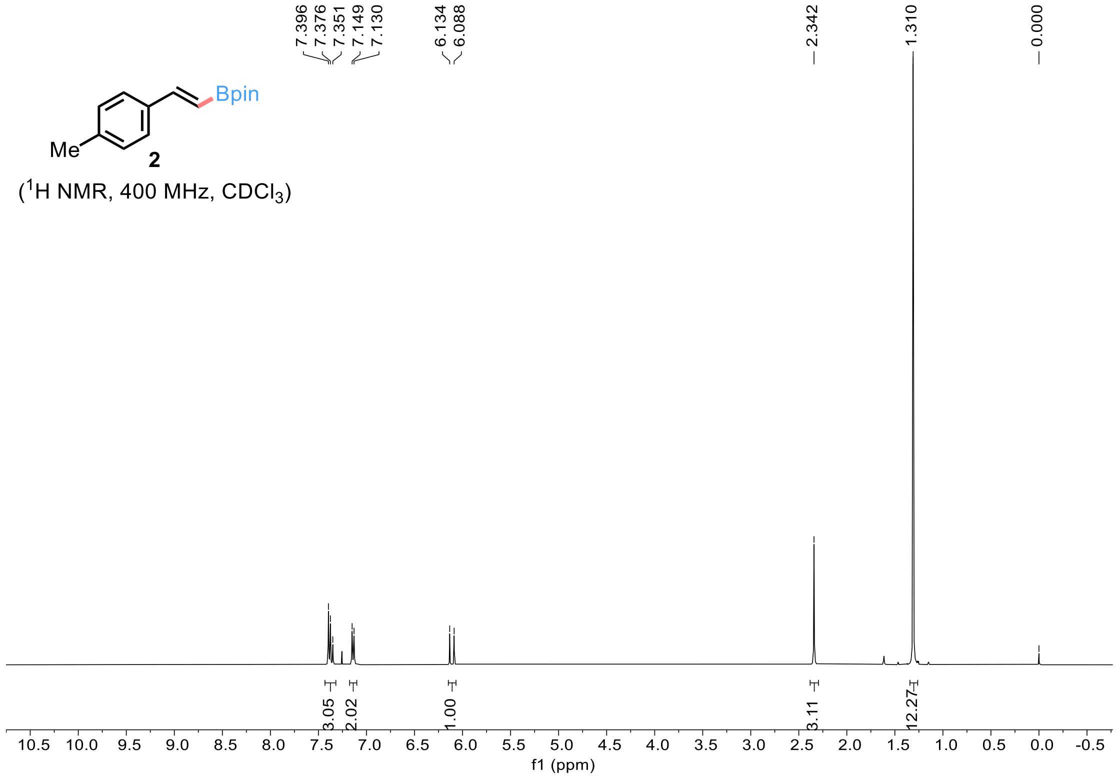
7.396
7.376
7.351
7.149
7.130
6.134
6.088
2.342
1.310
0.000
Bpin
Me
2
(¹H NMR, 400 MHz, CDCl3)
3.05
2.02
1.00
3.11
12.27
f1 (ppm)

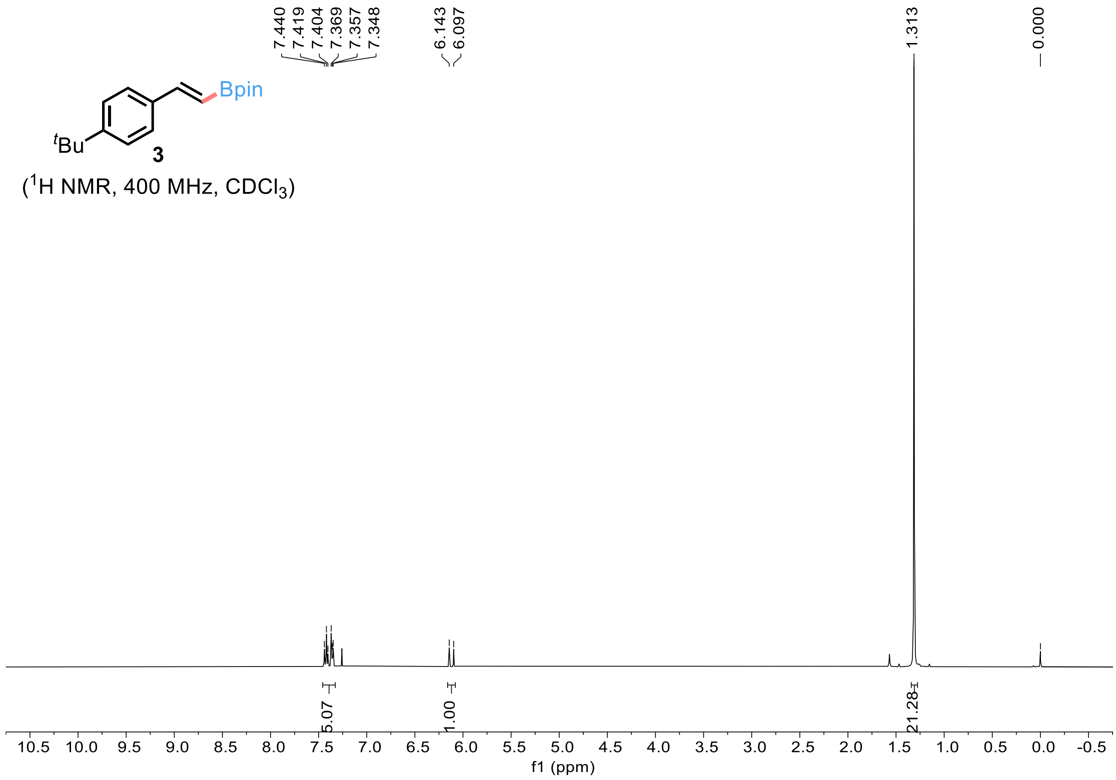

(^{1}H NMR, 400 MHz, $CDCl_3$)

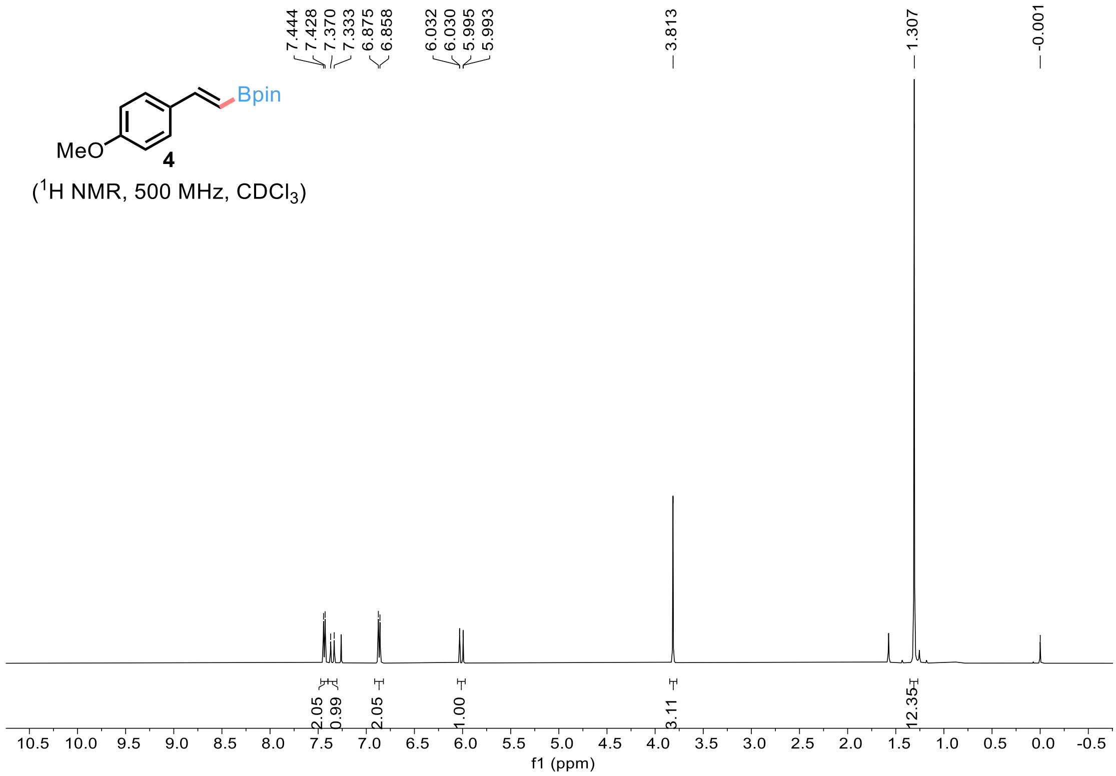

(^{1}H NMR, 500 MHz, $CDCl_3$)

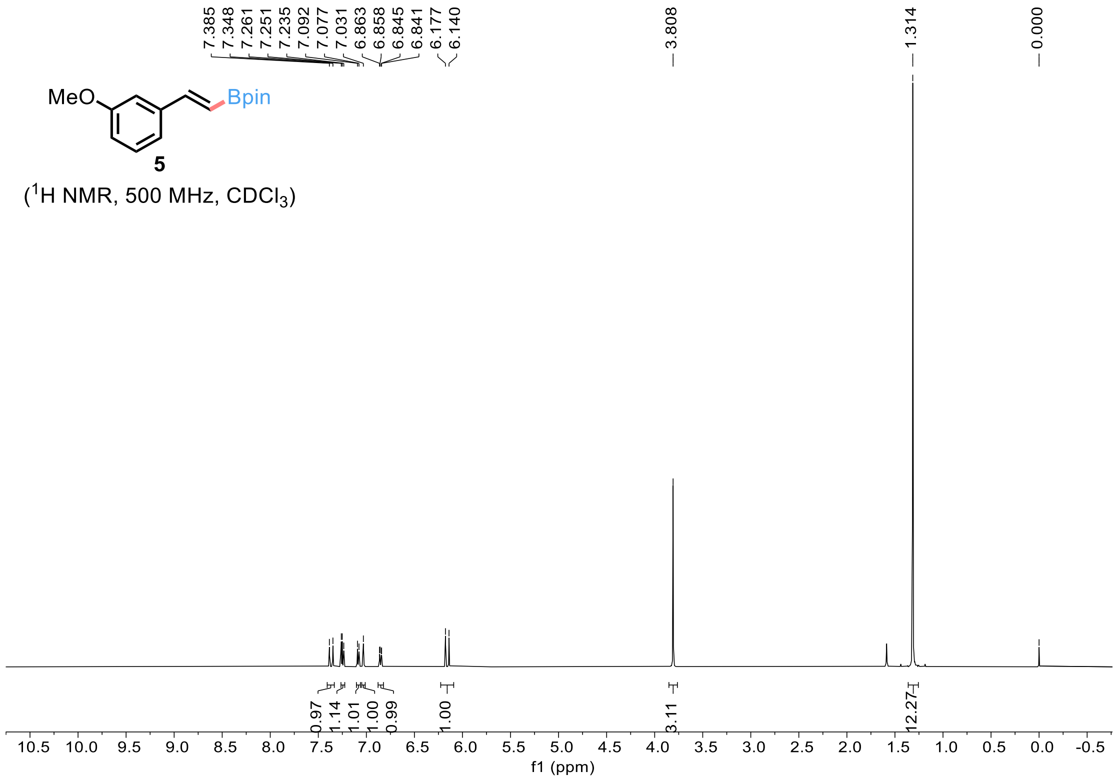

5

(^{1}H NMR, 500 MHz, $CDCl_3$)

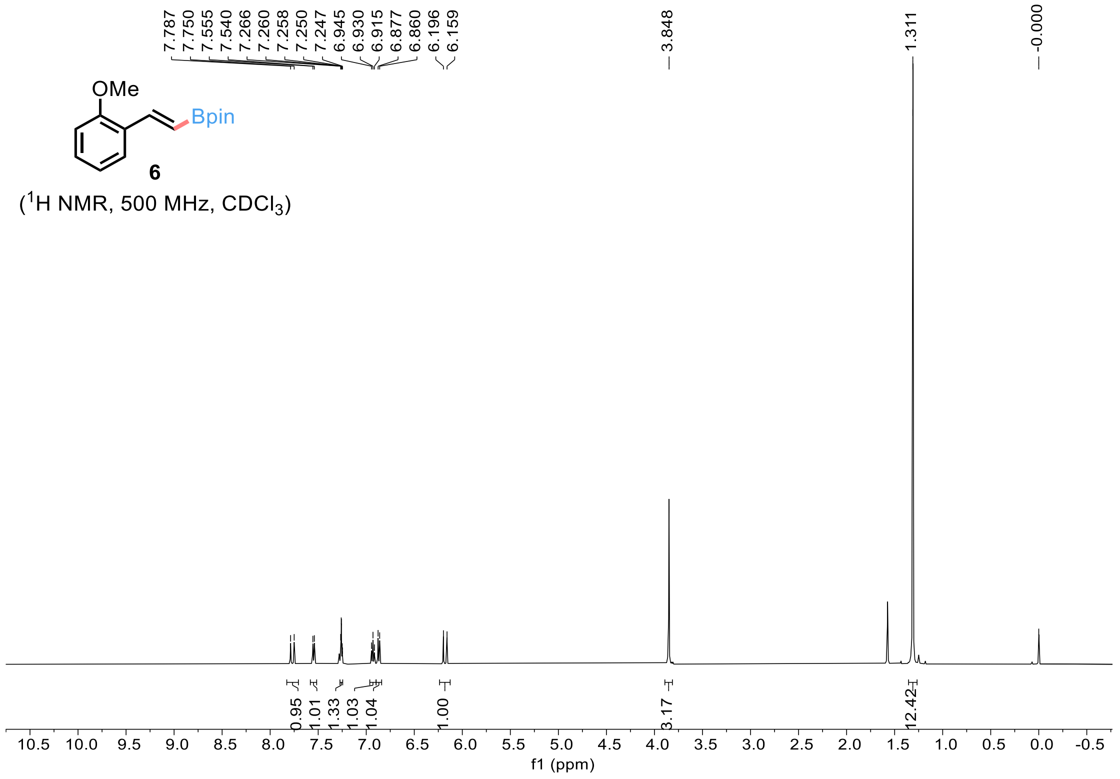

6

(^{1}H NMR, 500 MHz, $CDCl_3$)

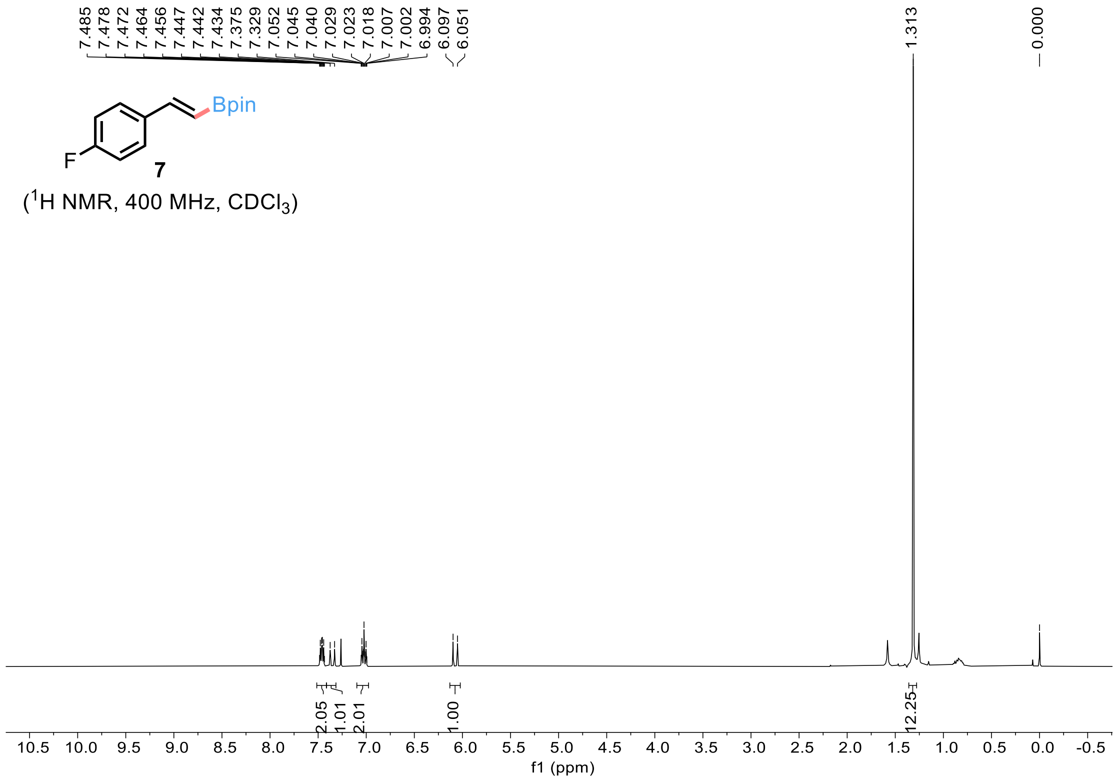

7

(^{1}H NMR, 400 MHz, $CDCl_3$)

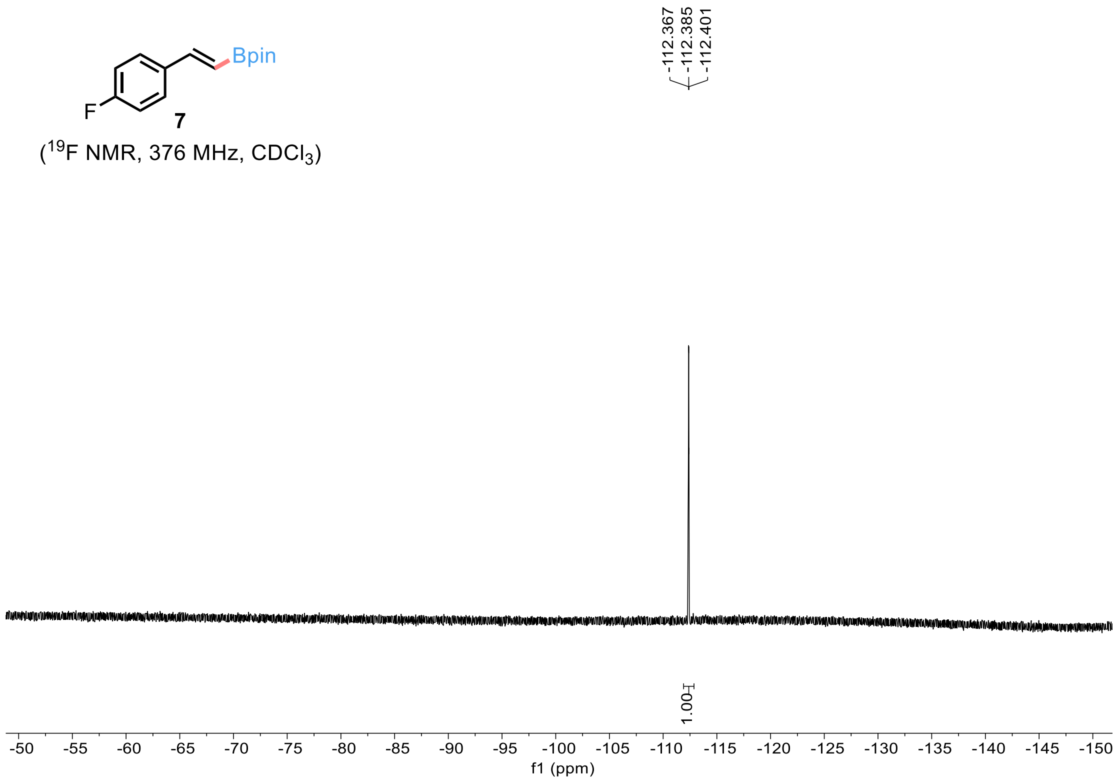

7

(^{19}F NMR, 376 MHz, $CDCl_3$)

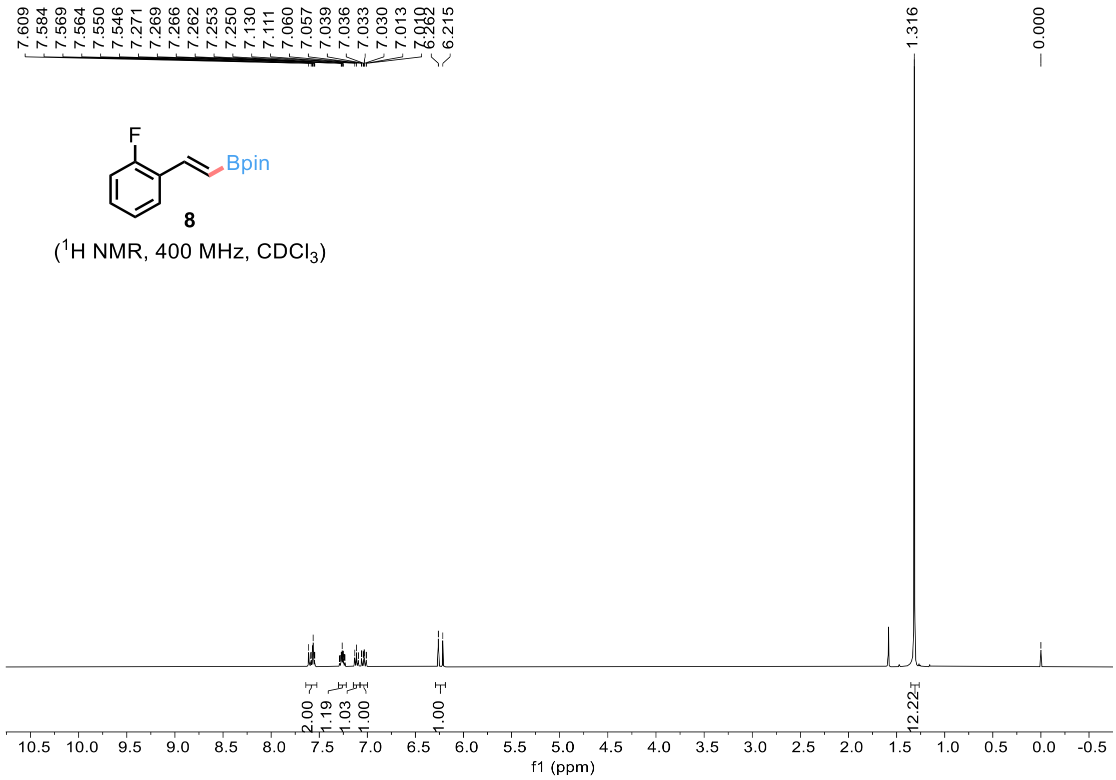
7.609
7.584
7.569
7.564
7.550
7.546
7.271
7.269
7.266
7.262
7.253
7.250
7.130
7.111
7.060
7.057
7.039
7.036
7.033
7.030
7.013
7.010
6.262
6.215
1.316
0.000
F
Bpin
8
(¹H NMR, 400 MHz, CDCl₃)
2.00
1.19
1.03
1.00
1.00
12.22
f1 (ppm)

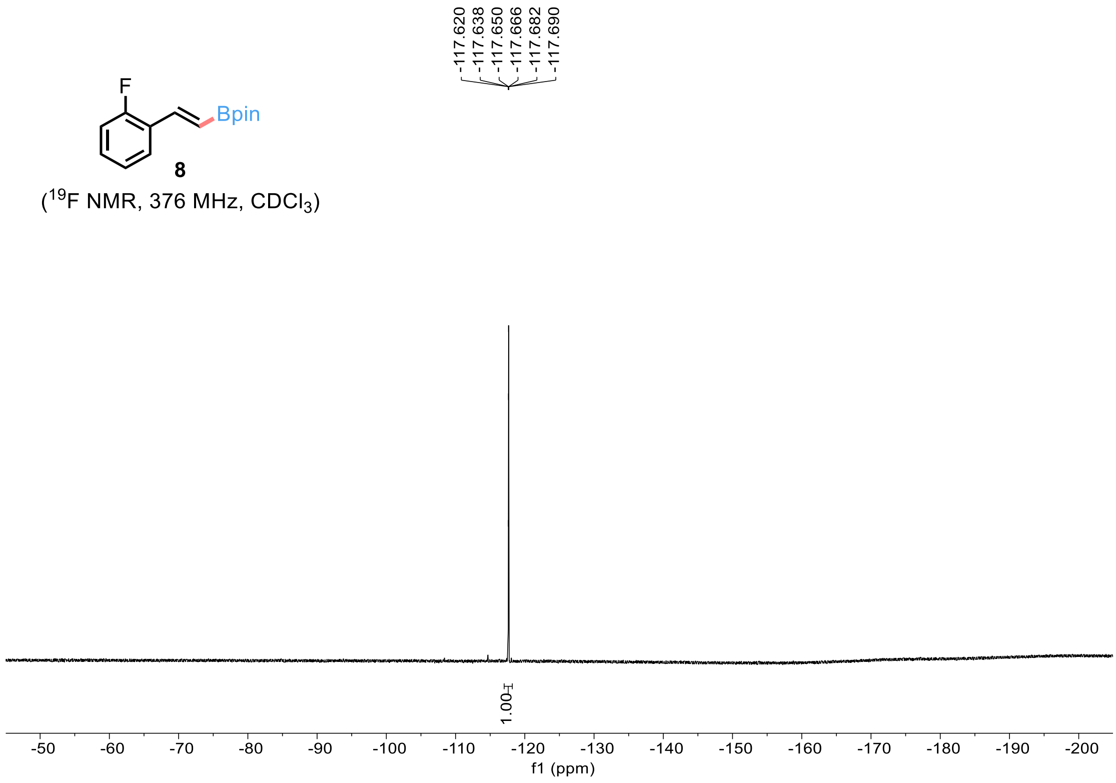
-117.620
-117.638
-117.650
-117.666
-117.682
-117.690
F
Bpin
8
(¹⁹F NMR, 376 MHz, CDCl₃)
1.00
f1 (ppm)

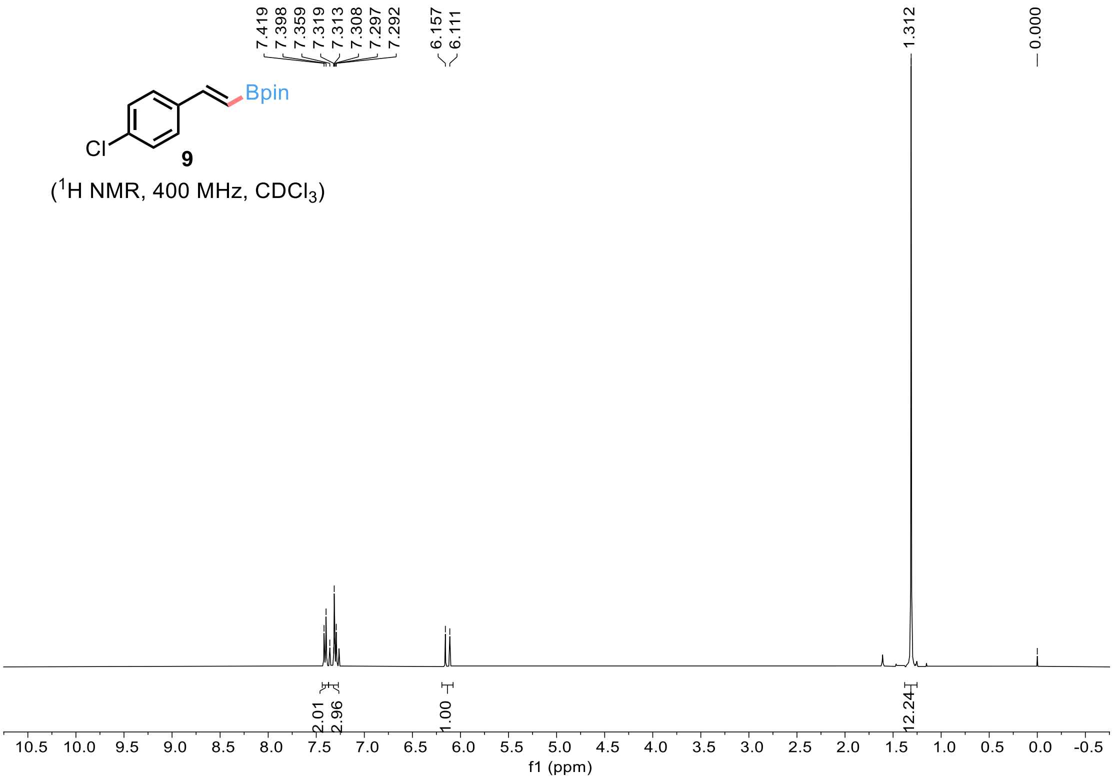
7.419
7.398
7.359
7.319
7.313
7.308
7.297
7.292
6.157
6.111
1.312
0.000
Bpin
Cl
9
(1H NMR, 400 MHz, CDCl3)
2.01
2.96
1.00
12.24
10.5 10.0 9.5 9.0 8.5 8.0 7.5 7.0 6.5 6.0 5.5 5.0 4.5 4.0 3.5 3.0 2.5 2.0 1.5 1.0 0.5 0.0 -0.5
f1 (ppm)

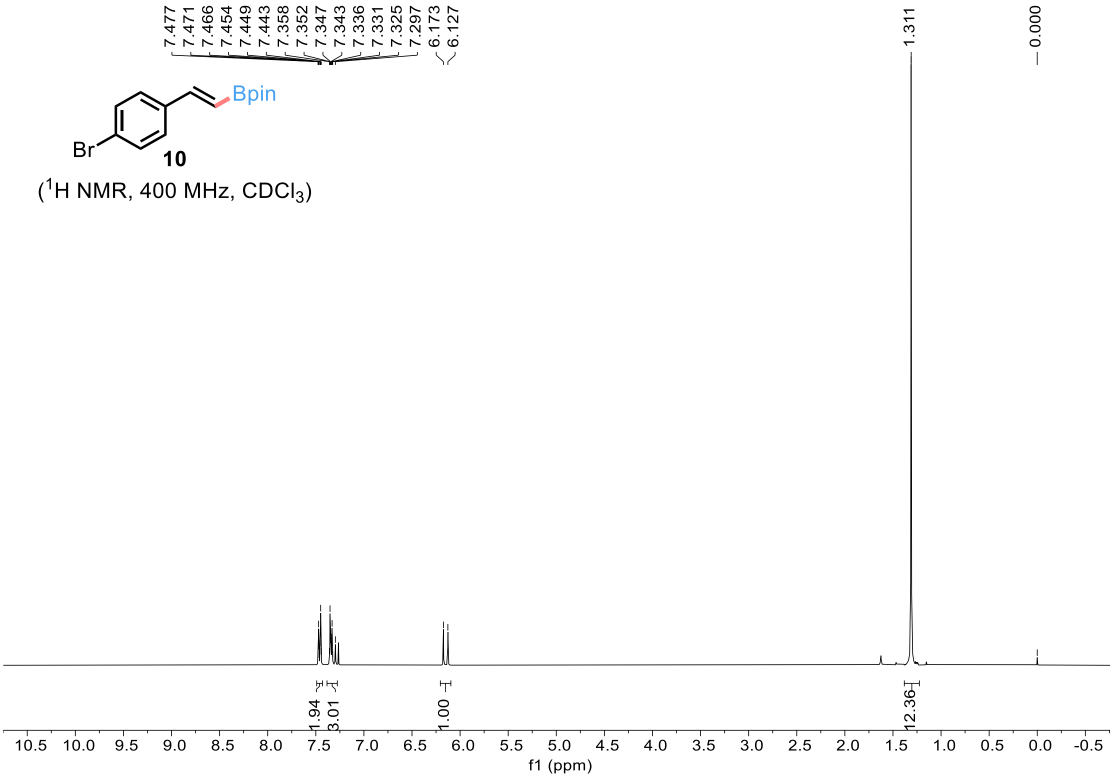
7.477
7.471
7.466
7.454
7.449
7.443
7.358
7.352
7.347
7.343
7.336
7.331
7.325
7.297
6.173
6.127
1.311
0.000
Bpin
Br
10
(1H NMR, 400 MHz, CDCl3)
1.94
3.01
1.00
12.36
10.5 10.0 9.5 9.0 8.5 8.0 7.5 7.0 6.5 6.0 5.5 5.0 4.5 4.0 3.5 3.0 2.5 2.0 1.5 1.0 0.5 0.0 -0.5
f1 (ppm)

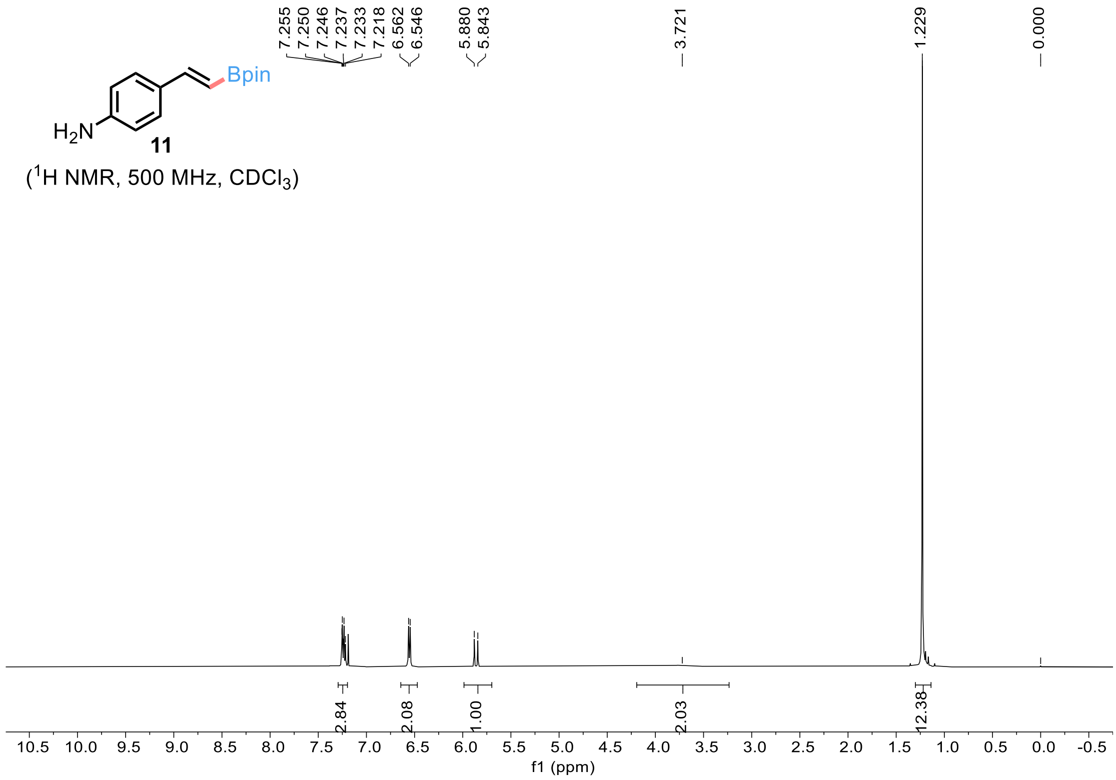

7.255
7.250
7.246
7.237
7.233
7.218
6.562
6.546
5.880
5.843
3.721
1.229
0.000
Bpin
H_2N
11
(^{1}H NMR, 500 MHz, $CDCl_3$)
2.84
2.08
1.00
2.03
12.38
10.5 10.0 9.5 9.0 8.5 8.0 7.5 7.0 6.5 6.0 5.5 5.0 4.5 4.0 3.5 3.0 2.5 2.0 1.5 1.0 0.5 0.0 -0.5
f1 (ppm)

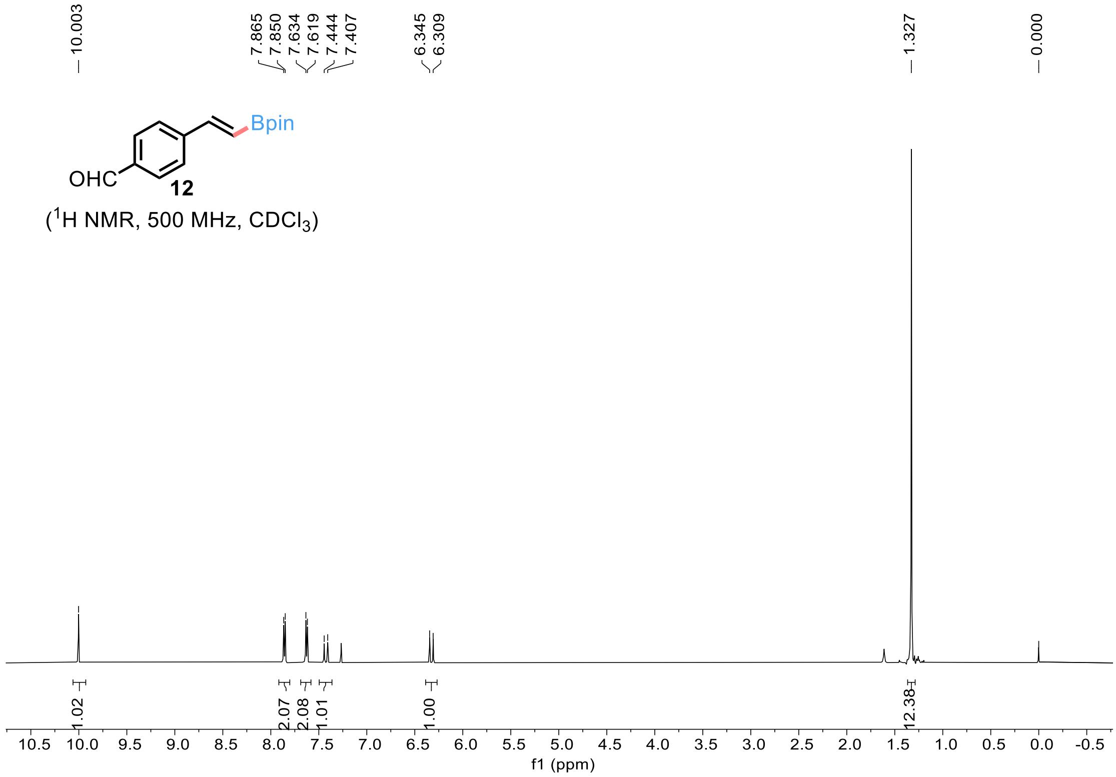

10.003
7.865
7.850
7.634
7.619
7.444
7.407
6.345
6.309
1.327
0.000
Bpin
OHC
12
(^{1}H NMR, 500 MHz, $CDCl_3$)
1.02
2.07
2.08
1.01
1.00
12.38
10.5 10.0 9.5 9.0 8.5 8.0 7.5 7.0 6.5 6.0 5.5 5.0 4.5 4.0 3.5 3.0 2.5 2.0 1.5 1.0 0.5 0.0 -0.5
f1 (ppm)

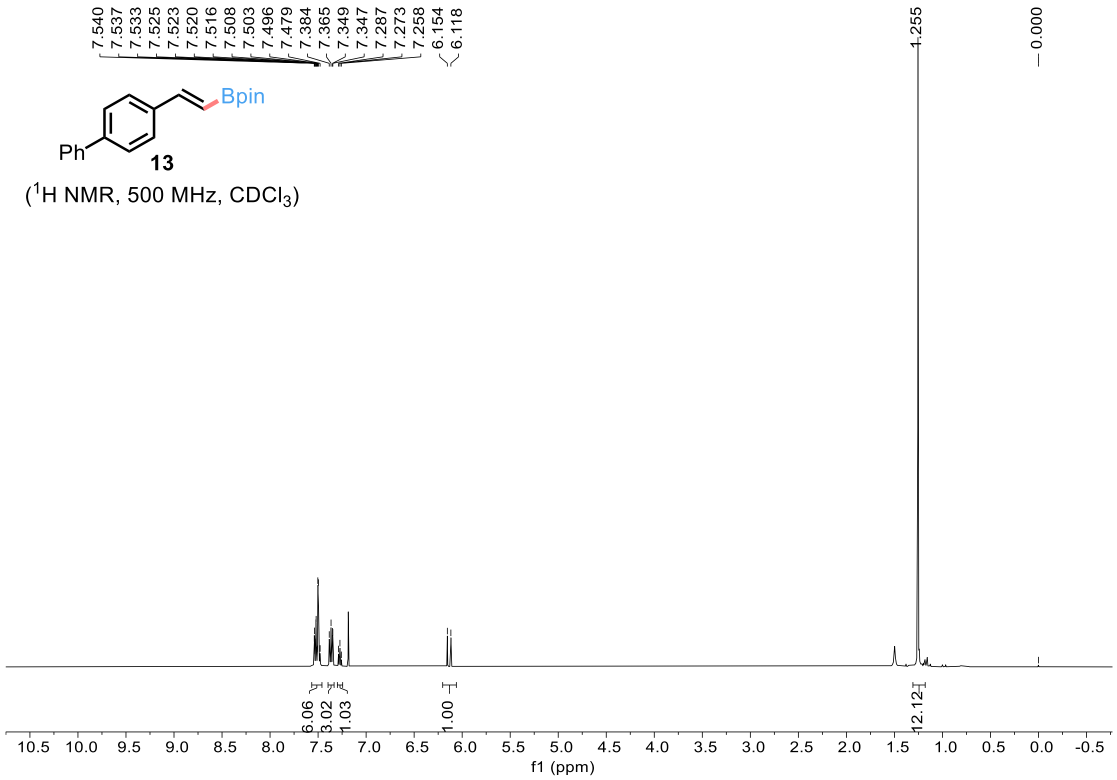

7.540
7.537
7.533
7.525
7.523
7.520
7.516
7.508
7.503
7.496
7.479
7.384
7.365
7.349
7.347
7.287
7.273
7.258
6.154
6.118
1.255
0.000
Bpin
Ph
13
(¹H NMR, 500 MHz, CDCl3)
6.06
3.02
1.03
1.00
12.12
f1 (ppm)

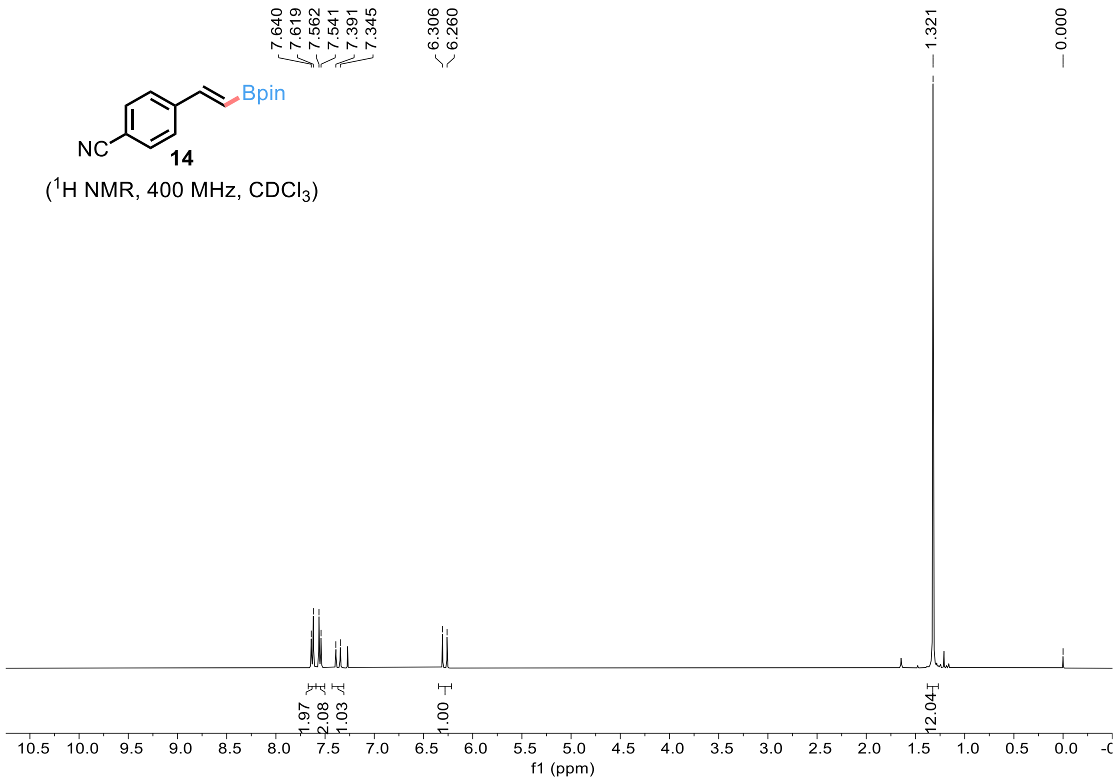

7.640
7.619
7.562
7.541
7.391
7.345
6.306
6.260
1.321
0.000
Bpin
NC
14
(¹H NMR, 400 MHz, CDCl3)
1.97
2.08
1.03
1.00
12.04
f1 (ppm)

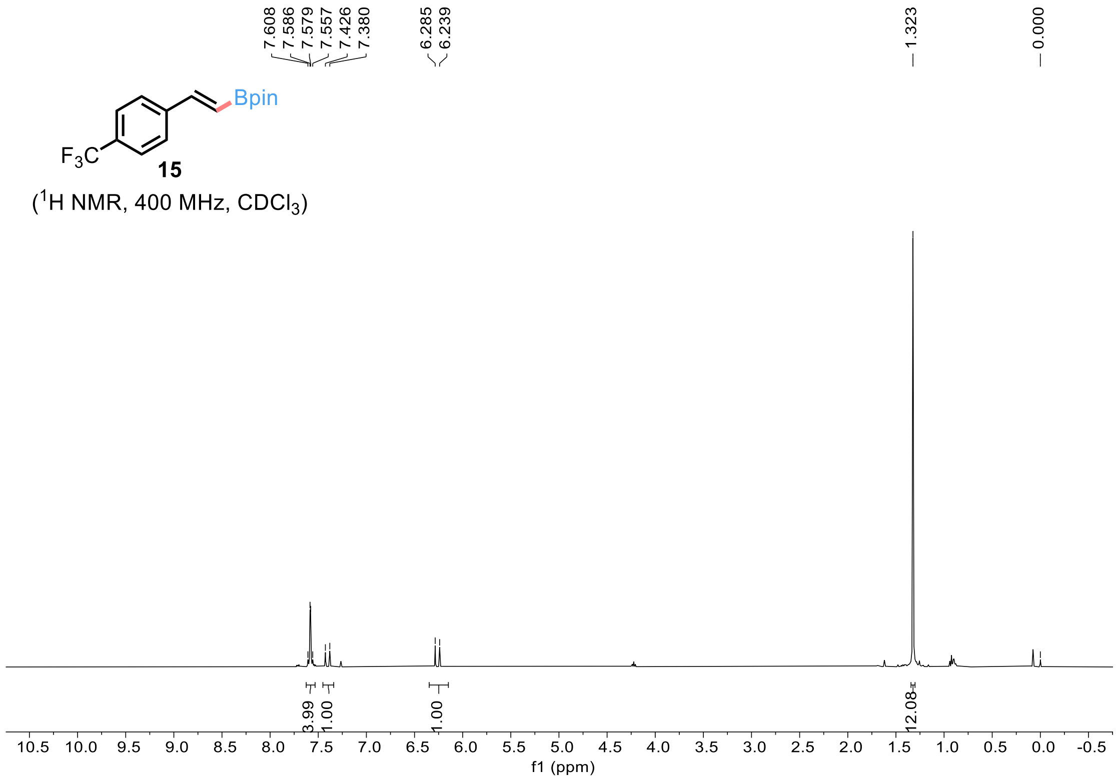
7.608
7.586
7.579
7.557
7.426
7.380
6.285
6.239
1.323
0.000
Bpin
F3C
15
(1H NMR, 400 MHz, CDCl3)
3.99
1.00
1.00
12.08
10.5 10.0 9.5 9.0 8.5 8.0 7.5 7.0 6.5 6.0 5.5 5.0 4.5 4.0 3.5 3.0 2.5 2.0 1.5 1.0 0.5 0.0 -0.5
f1 (ppm)

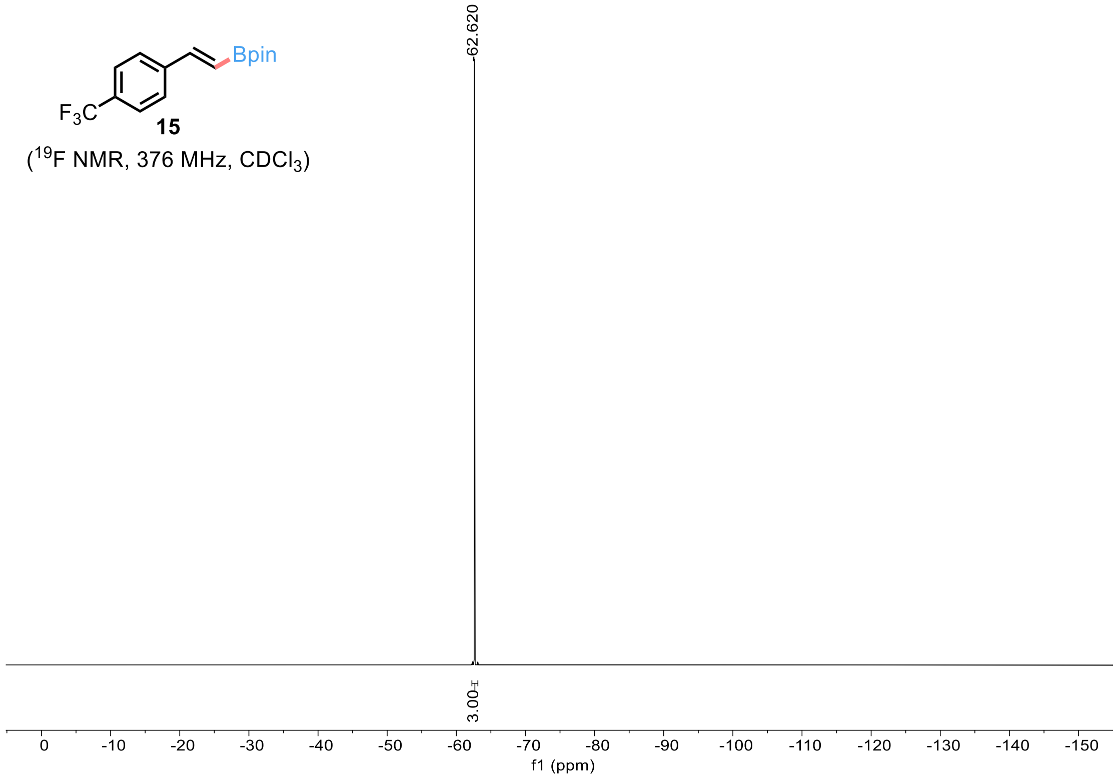
-62.620
Bpin
F3C
15
(19F NMR, 376 MHz, CDCl3)
3.00
0 -10 -20 -30 -40 -50 -60 -70 -80 -90 -100 -110 -120 -130 -140 -150
f1 (ppm)

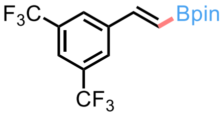

16

(^{1}H NMR, 400 MHz, $CDCl_3$)

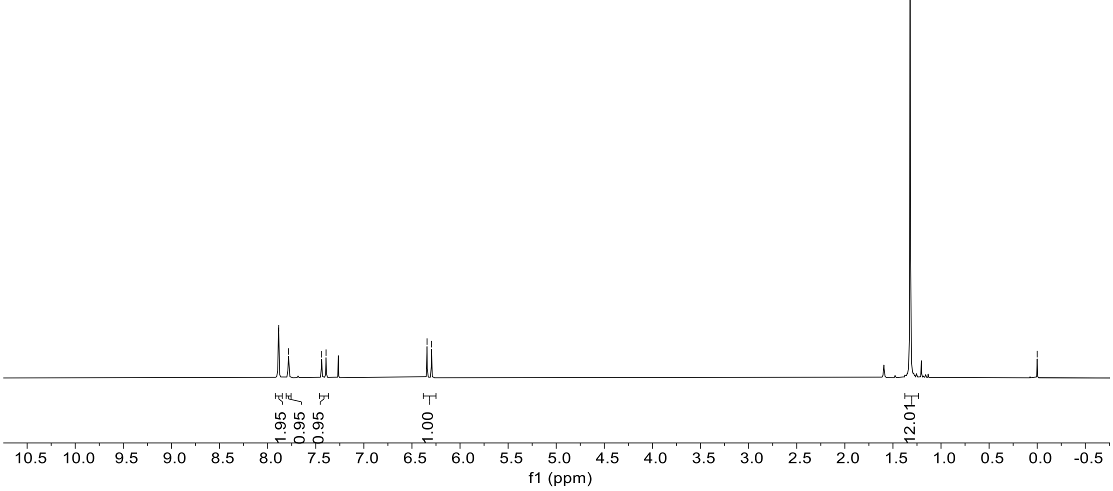

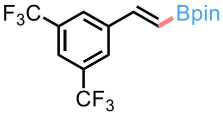

16

(^{19}F NMR, 376 MHz, $CDCl_3$)

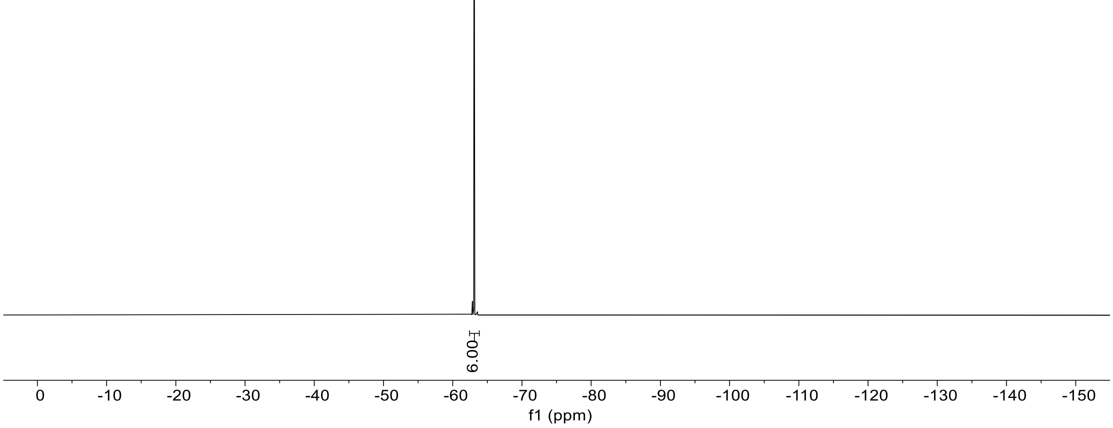

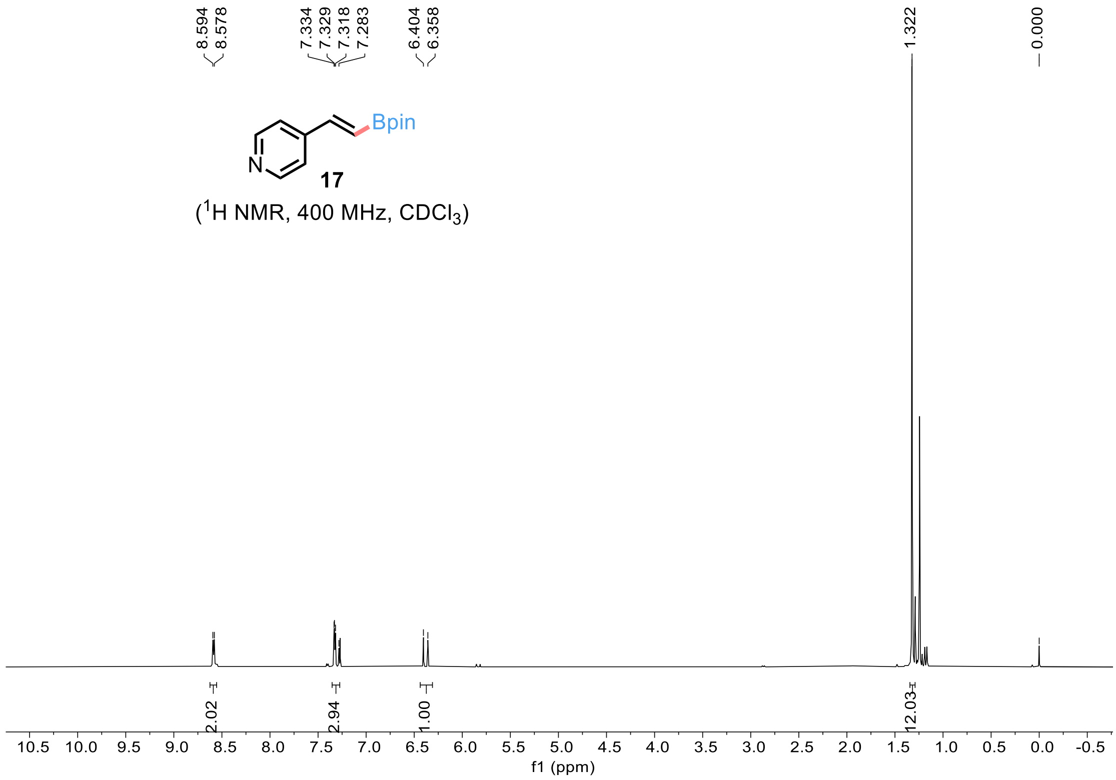
8.594
8.578
7.334
7.329
7.318
7.283
6.404
6.358
1.322
0.000
Bpin
N
17
(1H NMR, 400 MHz, CDCl3)
2.02
2.94
1.00
12.03
10.5 10.0 9.5 9.0 8.5 8.0 7.5 7.0 6.5 6.0 5.5 5.0 4.5 4.0 3.5 3.0 2.5 2.0 1.5 1.0 0.5 0.0 -0.5
f1 (ppm)

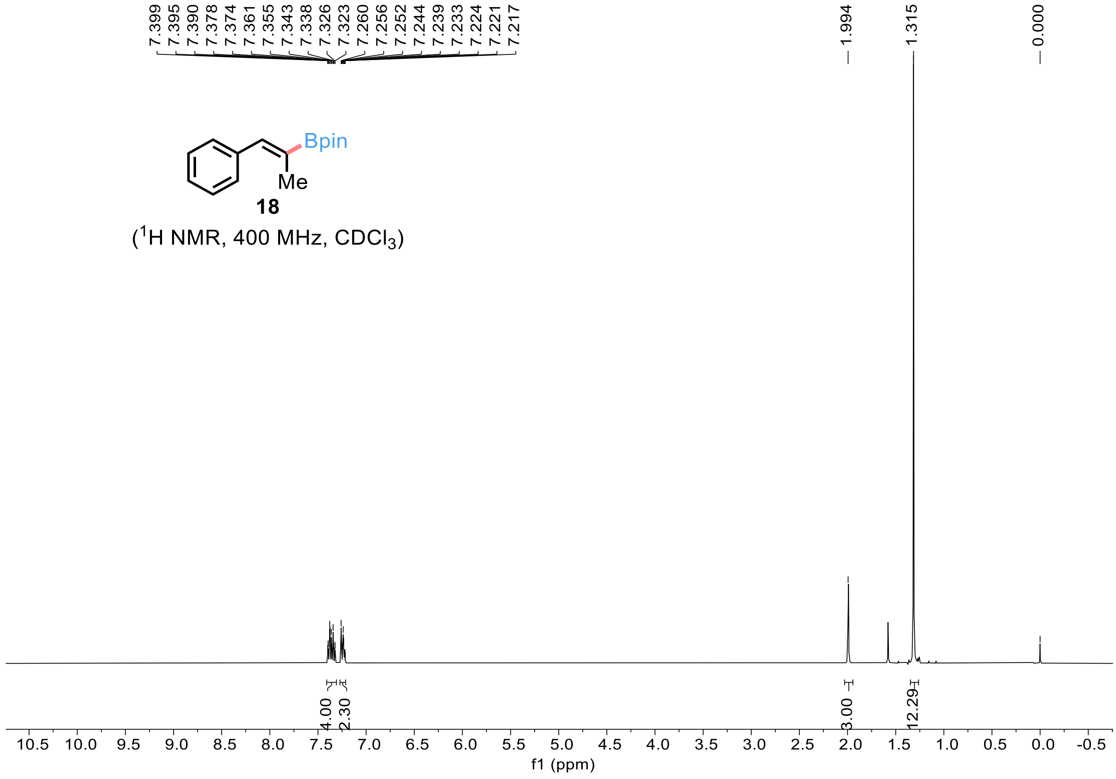
7.399
7.395
7.390
7.378
7.374
7.361
7.355
7.343
7.338
7.326
7.323
7.260
7.256
7.252
7.244
7.239
7.233
7.224
7.221
7.217
1.994
1.315
0.000
Bpin
Me
18
(1H NMR, 400 MHz, CDCl3)
4.00
2.30
3.00
12.29
10.5 10.0 9.5 9.0 8.5 8.0 7.5 7.0 6.5 6.0 5.5 5.0 4.5 4.0 3.5 3.0 2.5 2.0 1.5 1.0 0.5 0.0 -0.5
f1 (ppm)

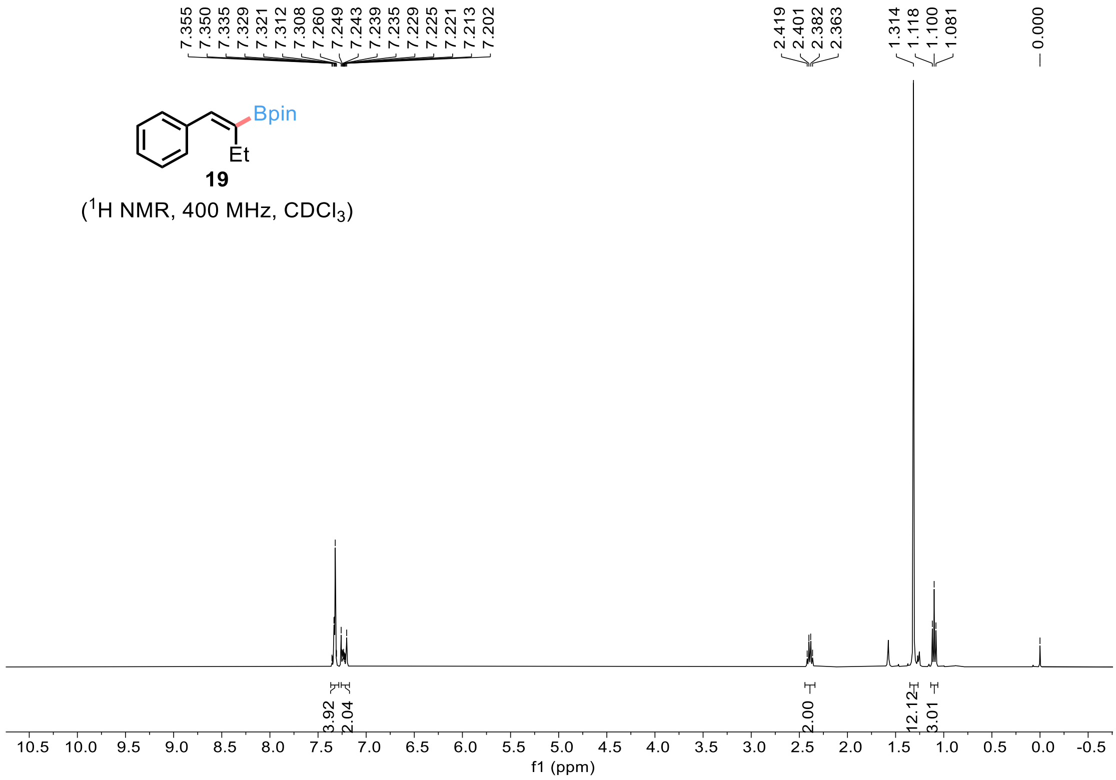
7.355 7.350 7.335 7.329 7.321 7.312 7.308 7.260 7.249 7.243 7.239 7.235 7.229 7.225 7.221 7.213 7.202
2.419 2.401 2.382 2.363
1.314 1.118 1.100 1.081
0.000
Bpin
Et
19
(1H NMR, 400 MHz, CDCl3)
3.92
2.04
2.00
12.12
3.01
10.5 10.0 9.5 9.0 8.5 8.0 7.5 7.0 6.5 6.0 5.5 5.0 4.5 4.0 3.5 3.0 2.5 2.0 1.5 1.0 0.5 0.0 -0.5
f1 (ppm)

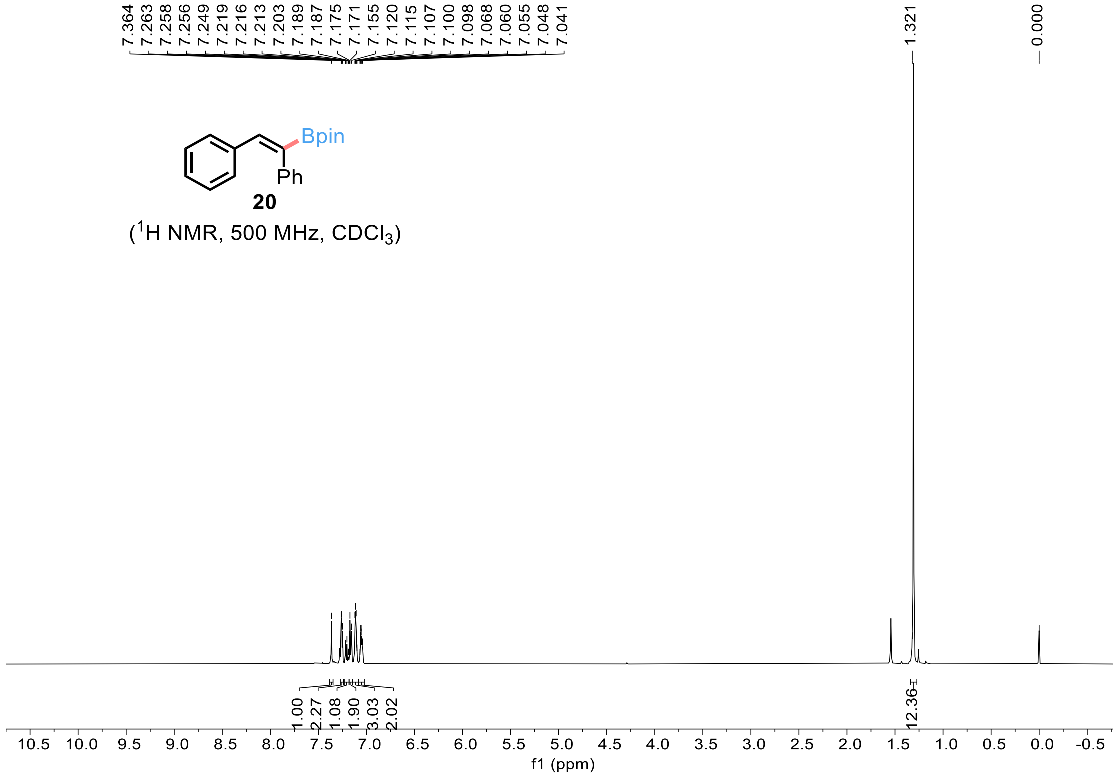
7.364 7.263 7.258 7.256 7.249 7.219 7.216 7.213 7.203 7.189 7.187 7.175 7.171 7.155 7.120 7.115 7.107 7.100 7.098 7.068 7.060 7.055 7.048 7.041
1.321
0.000
Bpin
Ph
20
(1H NMR, 500 MHz, CDCl3)
1.00
2.27
1.08
1.90
3.03
2.02
12.36
10.5 10.0 9.5 9.0 8.5 8.0 7.5 7.0 6.5 6.0 5.5 5.0 4.5 4.0 3.5 3.0 2.5 2.0 1.5 1.0 0.5 0.0 -0.5
f1 (ppm)

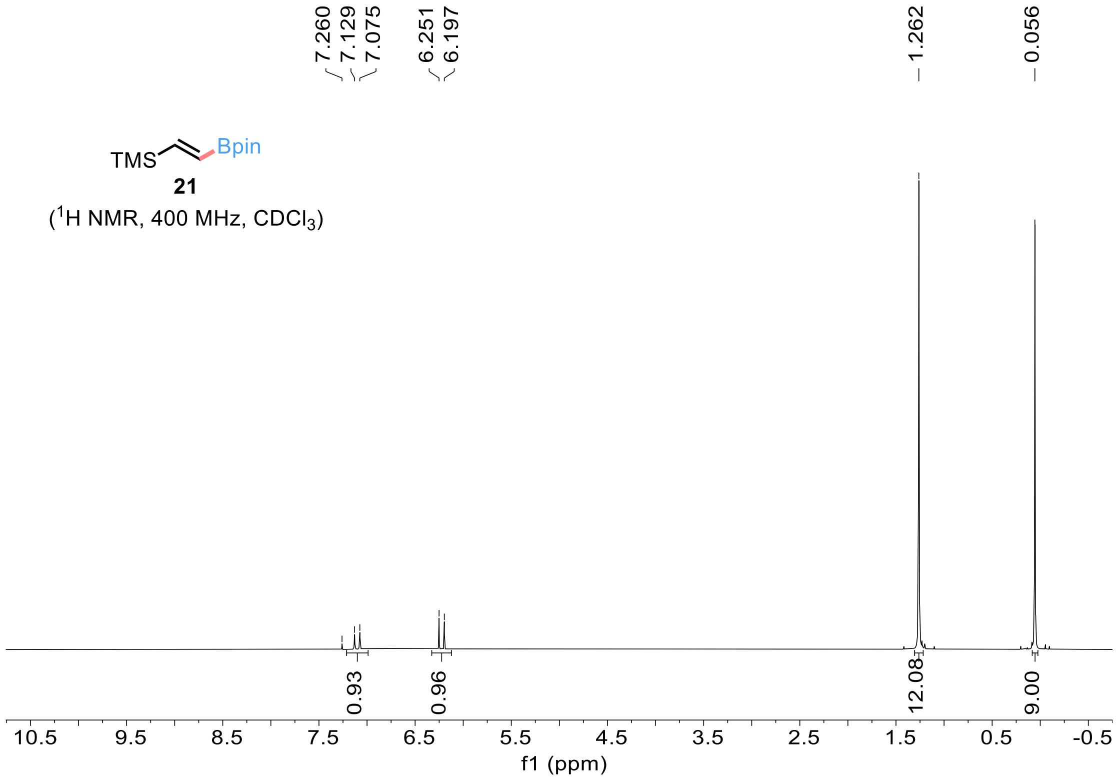
7.260
7.129
7.075
6.251
6.197
1.262
0.056
TMS
Bpin
21
(1H NMR, 400 MHz, CDCl3)
0.93
0.96
12.08
9.00
10.5 9.5 8.5 7.5 6.5 5.5 4.5 3.5 2.5 1.5 0.5 -0.5
f1 (ppm)

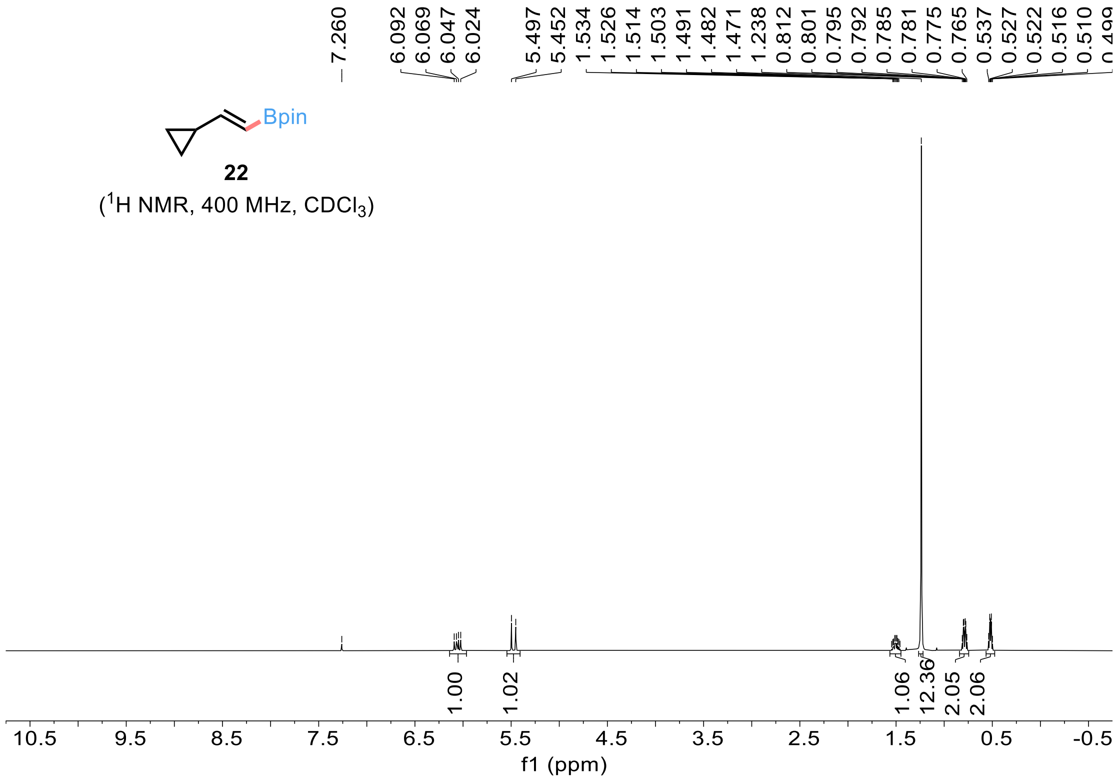
7.260
6.092
6.069
6.047
6.024
5.497
5.452
1.534
1.526
1.514
1.503
1.491
1.482
1.471
1.238
0.812
0.801
0.795
0.792
0.785
0.781
0.775
0.765
0.537
0.527
0.522
0.516
0.510
Bpin
22
(1H NMR, 400 MHz, CDCl3)
1.00
1.02
1.06
12.36
2.05
2.06
10.5 9.5 8.5 7.5 6.5 5.5 4.5 3.5 2.5 1.5 0.5 -0.5
f1 (ppm)

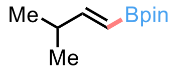

23

(^{1}H NMR, 400 MHz, $CDCl_3$)

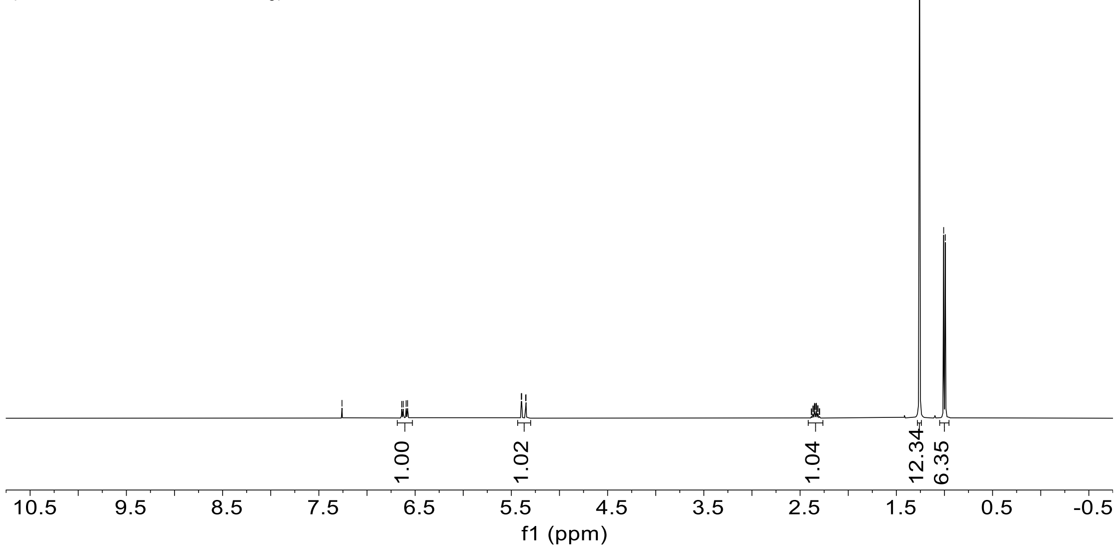

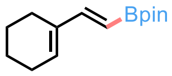

24

(^{1}H NMR, 400 MHz, $CDCl_3$)

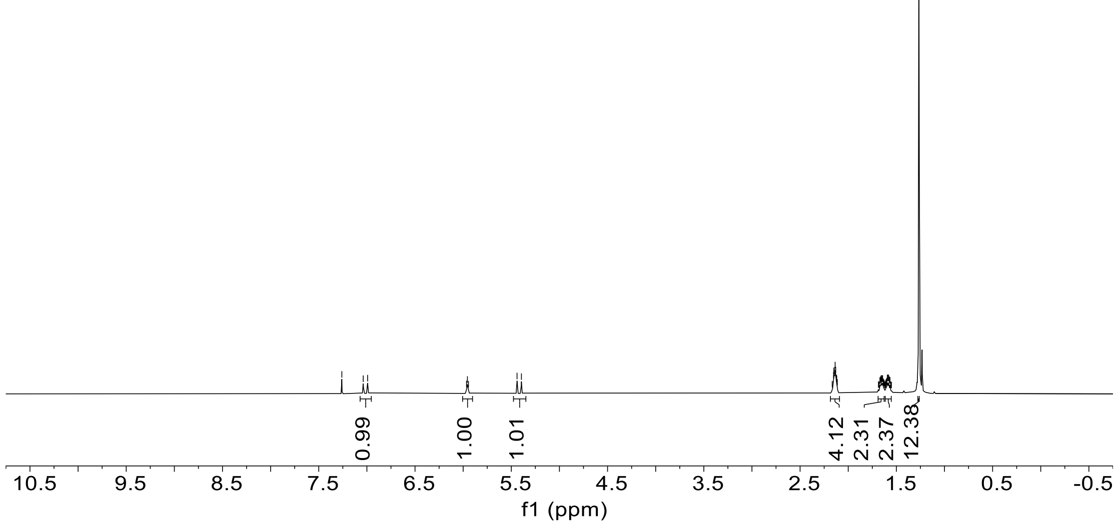

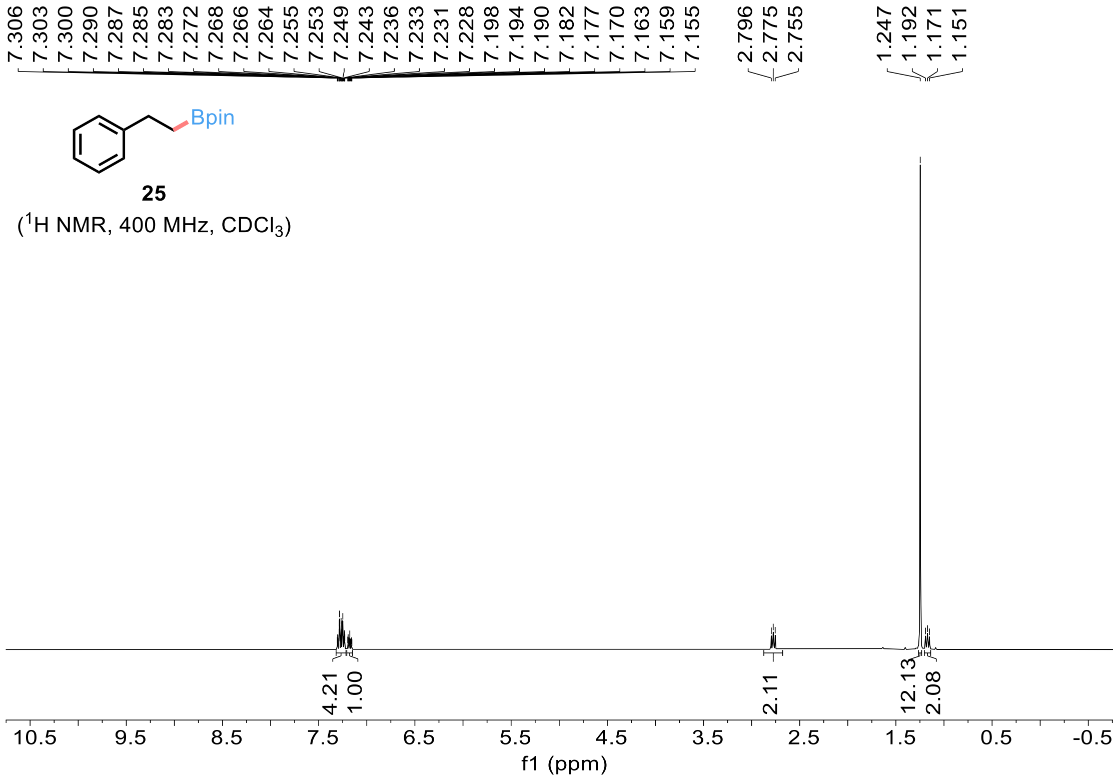
7.306 7.303 7.300 7.290 7.287 7.285 7.283 7.272 7.268 7.266 7.264 7.255 7.253 7.249 7.243 7.236 7.233 7.231 7.228 7.198 7.194 7.190 7.182 7.177 7.170 7.163 7.159 7.155
2.796 2.775 2.755
1.247 1.192 1.171 1.151
Bpin
25
(1H NMR, 400 MHz, CDCl3)
4.21
1.00
2.11
12.13
2.08
10.5 9.5 8.5 7.5 6.5 5.5 4.5 3.5 2.5 1.5 0.5 -0.5
f1 (ppm)

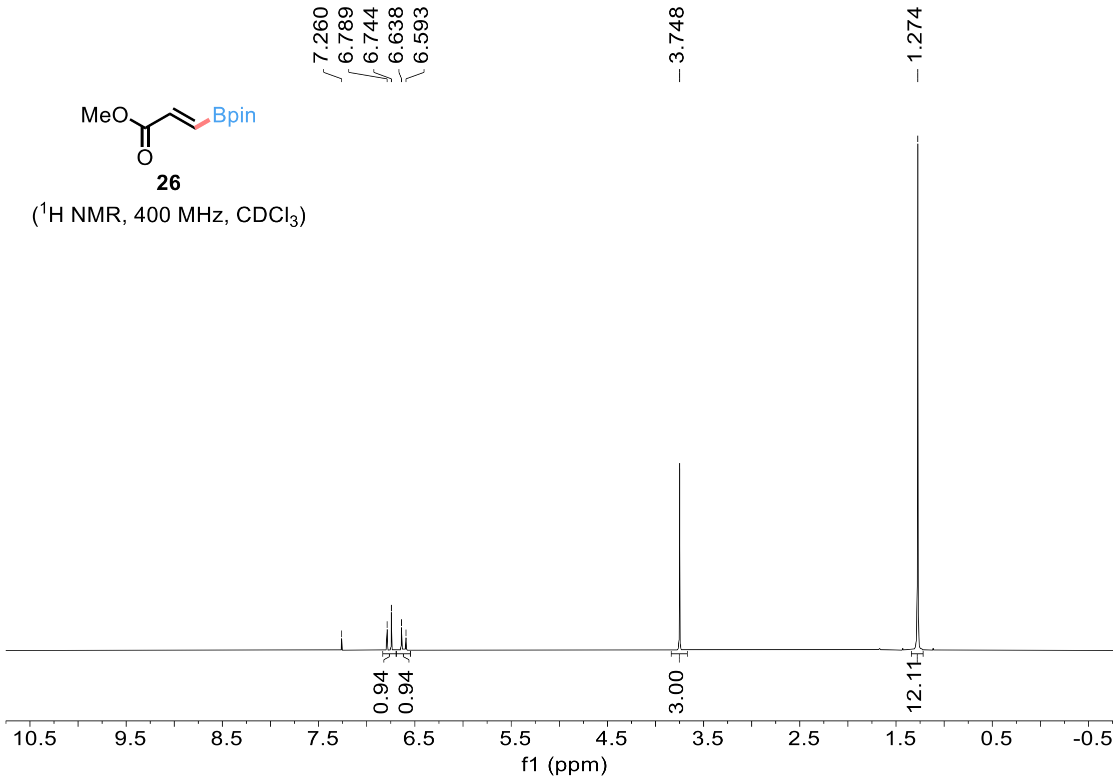
7.260 6.789 6.744 6.638 6.593
3.748
1.274
MeO
Bpin
O
26
(1H NMR, 400 MHz, CDCl3)
0.94
0.94
3.00
12.11
10.5 9.5 8.5 7.5 6.5 5.5 4.5 3.5 2.5 1.5 0.5 -0.5
f1 (ppm)

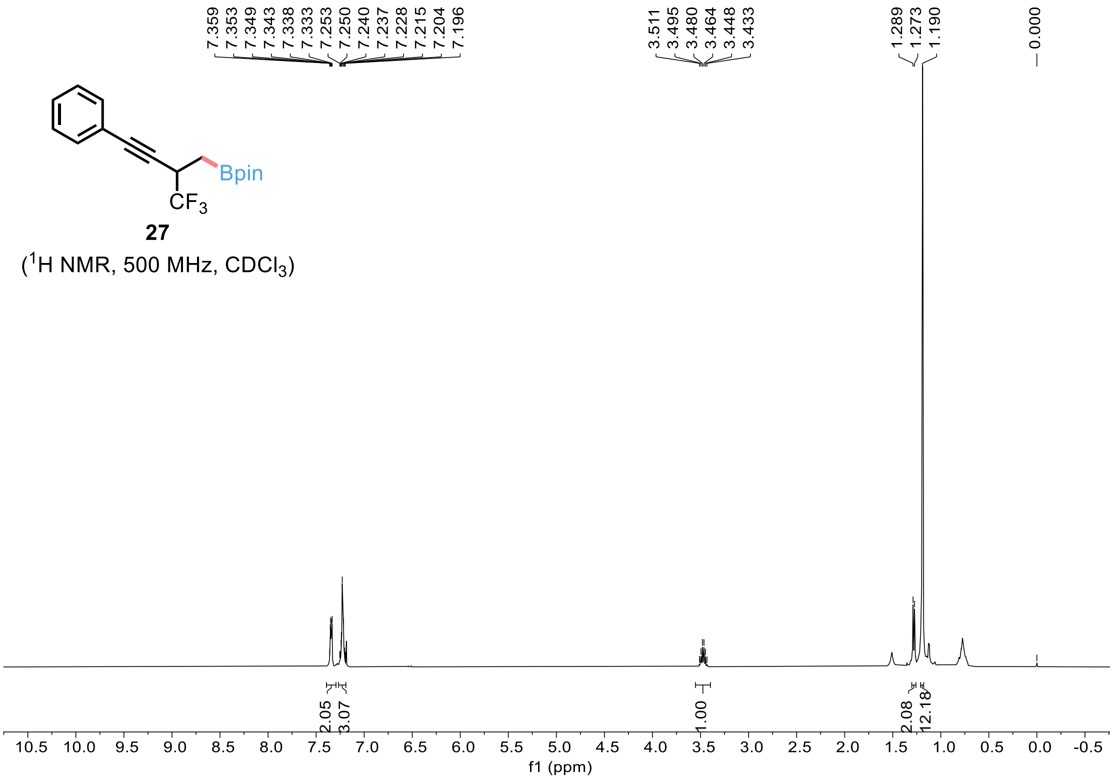
7.359
7.353
7.349
7.343
7.338
7.333
7.253
7.250
7.240
7.237
7.228
7.215
7.204
7.196
3.511
3.495
3.480
3.464
3.448
3.433
1.289
1.273
1.190
0.000
Bpin
CF3
27
(1H NMR, 500 MHz, CDCl3)
2.05
3.07
1.00
2.08
12.18
10.5 10.0 9.5 9.0 8.5 8.0 7.5 7.0 6.5 6.0 5.5 5.0 4.5 4.0 3.5 3.0 2.5 2.0 1.5 1.0 0.5 0.0 -0.5
f1 (ppm)

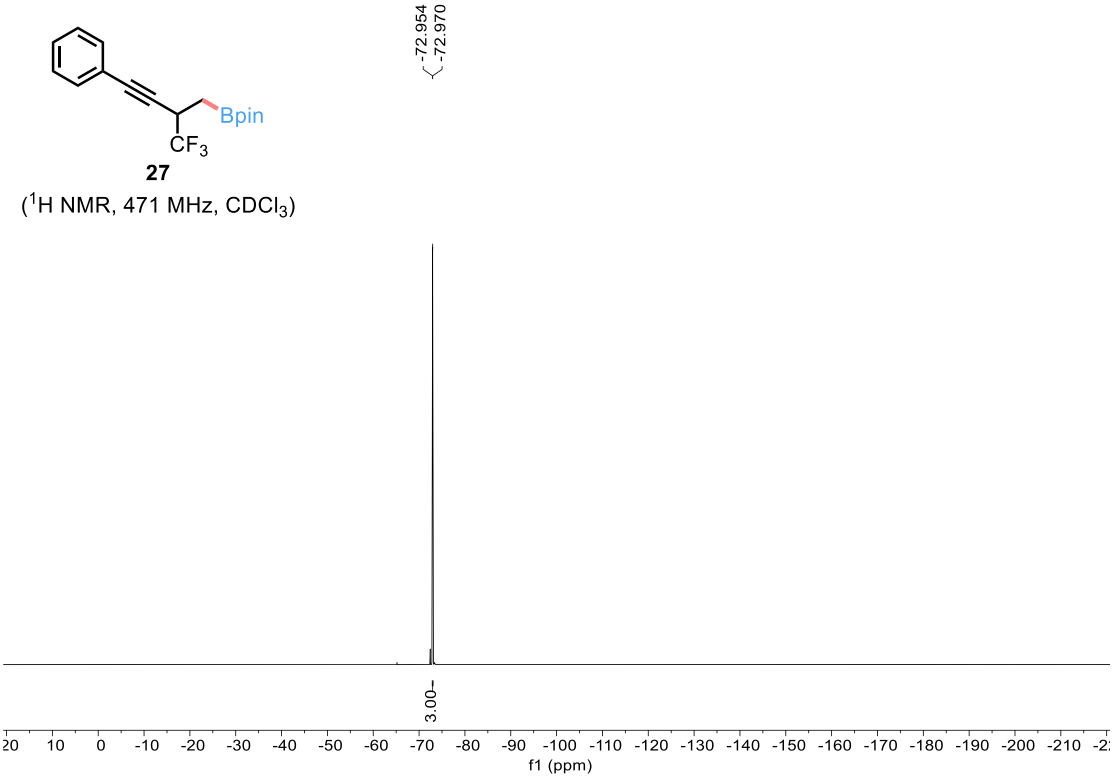
-72.954
-72.970
Bpin
CF3
27
(1H NMR, 471 MHz, CDCl3)
3.00
20 10 0 -10 -20 -30 -40 -50 -60 -70 -80 -90 -100 -110 -120 -130 -140 -150 -160 -170 -180 -190 -200 -210
f1 (ppm)

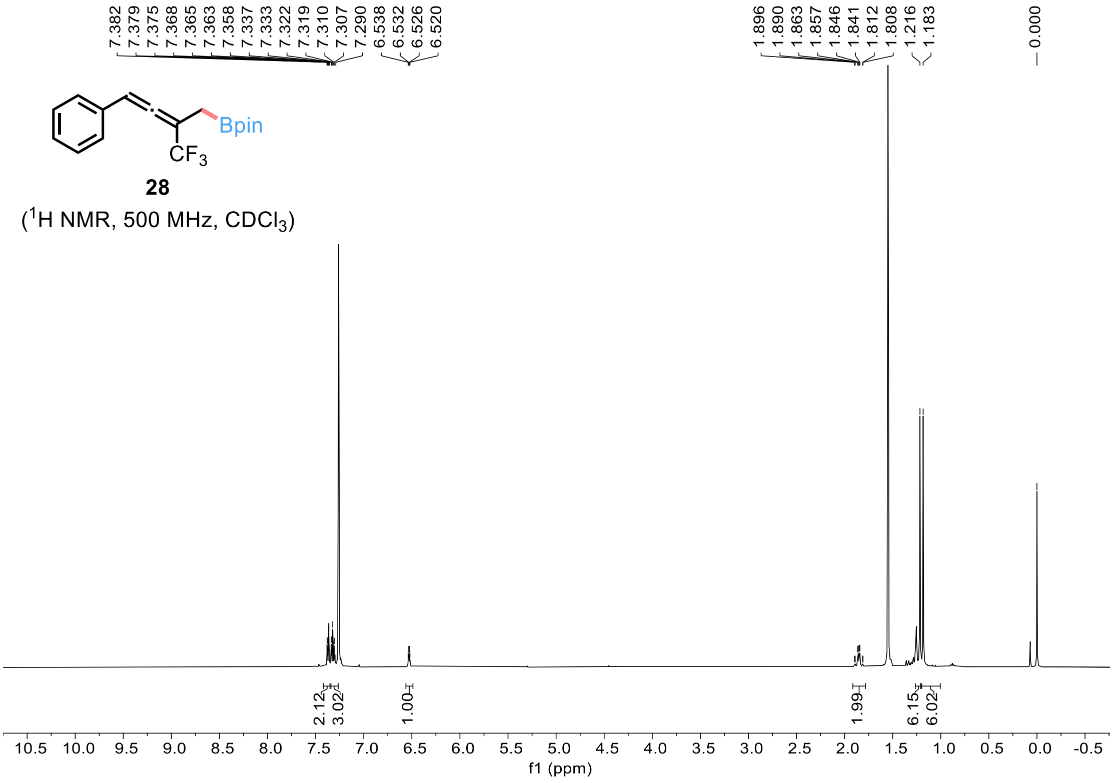
7.382
7.379
7.375
7.368
7.365
7.363
7.358
7.337
7.333
7.322
7.319
7.310
7.307
7.290
6.538
6.532
6.526
6.520
1.896
1.890
1.863
1.857
1.846
1.841
1.812
1.808
1.216
1.183
0.000
Bpin
CF_3
28
(^1H NMR, 500 MHz, $CDCl_3$)
2.12
3.02
1.00
1.99
6.15
6.02
10.5 10.0 9.5 9.0 8.5 8.0 7.5 7.0 6.5 6.0 5.5 5.0 4.5 4.0 3.5 3.0 2.5 2.0 1.5 1.0 0.5 0.0 -0.5
f1 (ppm)

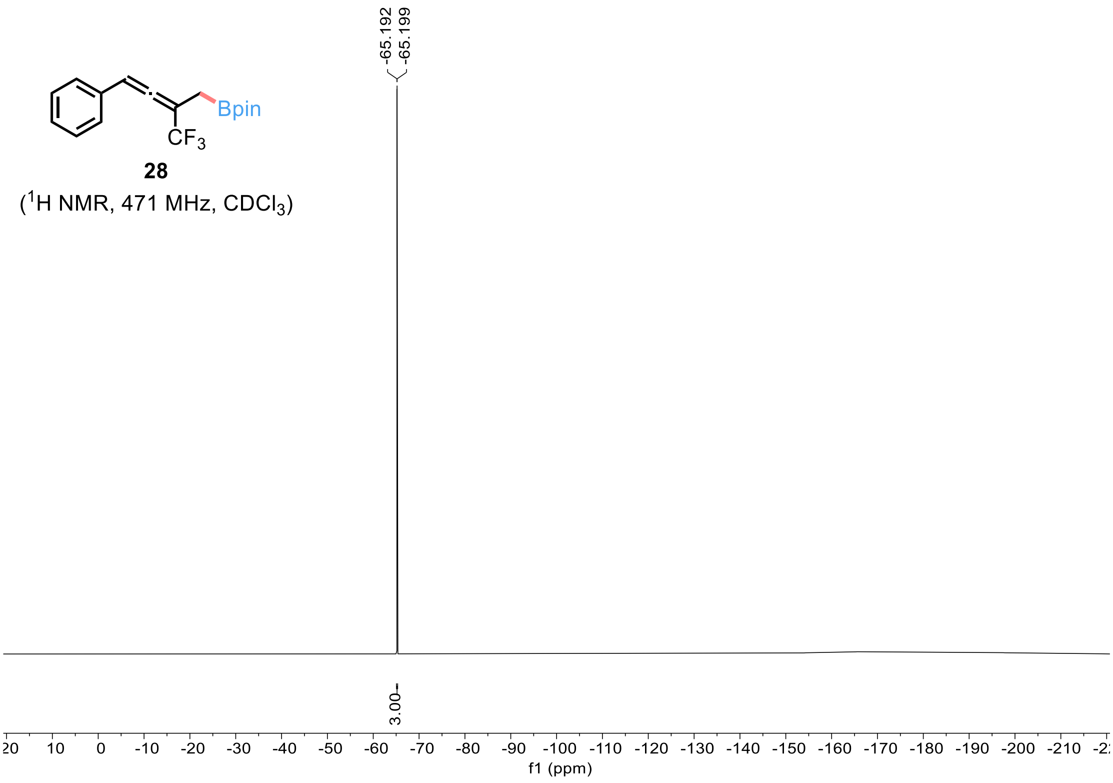
-65.192
-65.199
Bpin
CF_3
28
(^1H NMR, 471 MHz, $CDCl_3$)
3.00
20 10 0 -10 -20 -30 -40 -50 -60 -70 -80 -90 -100 -110 -120 -130 -140 -150 -160 -170 -180 -190 -200 -210
f1 (ppm)
